# Impact of Design Parameters on the Ratio of Compressive to Split Tensile Strength of Self-Compacting Concrete with Recycled Aggregate

**DOI:** 10.3390/ma14133480

**Published:** 2021-06-22

**Authors:** Rebeca Martínez-García, P. Jagadesh, Gabriel Búrdalo-Salcedo, Covadonga Palencia, María Fernández-Raga, Fernando J. Fraile-Fernández

**Affiliations:** 1 Department of Mining Technology, Topography and Structures, Campus de Vegazana s/n, University of León, 24071 León, Spain; fjfraf@unileon.es; 2 Department of Civil Engineering, Coimbatore Institute of Technology, Coimbatore 641014, Tamil Nadu, India; 3 Department of Applied Physics, Campus de Vegazana s/n, University of León, 24071 León, Spain; gabriel.burdalo@unileon.es (G.B.-S.); c.palencia@unileon.es (C.P.); mferr@unileon.es (M.F.-R.)

**Keywords:** strength ratio, self-compacting concrete, recycled aggregates, design parameters

## Abstract

Most concrete studies are concentrated on mechanical properties especially strength properties either directly or indirectly (fresh and durability properties). Hence, the ratio of split tensile strength to compressive strength plays a vital role in defining the concrete properties. In this review, the impact of design parameters on the strength ratio of various grades of Self-Compacting Concrete (SCC) with recycled aggregate is assessed. The design parameters considered for the study are Water to Cement (W/C) ratio, Water to Binder (W/B) ratio, Total Aggregates to Cement (TA/C) ratio, Fine Aggregate to Coarse Aggregate (FA/CA) ratio, Water to Solid (W/S) ratio in percentage, superplasticizer (SP) content (kg/cu.m), replacement percentage of recycled coarse aggregates (RCA), replacement percentage of recycled fine aggregates (RFA), fresh density and loading area of the specimen. It is observed that the strength ratio of SCC with recycled aggregates is affected by design parameters.

## 1. Introduction

In recent days, the boom in urban development and industrialization has resulted in the high consumption of natural aggregates apart from other materials. And also, due to demolition of the second largest consumption material (concrete) by humans, leads to the generation of construction and demolition waste (C&DW), which is to be relocated, reused, or recycled. The constant population growth and modernization of regions imply a daily increase in the generation of concrete waste [1]. Researchers estimated that aggregate consumption, the global consumption of aggregate for construction, is expected to reach 62.9 billion metric tonnes by the end of 2024, up from 43.3 billion metric tonnes in 2016, in terms of volume. The damage to ecosystems could be reversed with the classification of waste and residues, therefore, the aggregates used for construction, preparing concrete or other types of mixtures, would have a more sustainable purpose. For a country, one of the main investment sectors across the industries available is the construction sector [2]. Concrete is one of the factors that determines the level of development of countries, not only for the construction of new civil/architectural works but also playing an important role in the repair, retrofitting, and/or reconstruction of existing works.

According to data from the European Statistical Office, Eurostat, each European citizen produces an average of 2000 kg of waste per year, excluding mining waste (including the latter, this figure would exceed 5000 kg/person/year) [3]. Of this waste pool, more than one third corresponds to the construction sector. For this reason, the study of the use of this demolition waste has been reinforced by researchers around the world, and even countries such as Italy and Denmark are developing standards and principles related to its processing [4,5,6]. There are few countries (such as Denmark and Germany) which have achieved reuse of demolition waste in higher percentages than 80% [7]. On the other hand, other nations have reported percentages lower than 10% [8]. This is the reason why the recycling issue is still in force. The use of recycled aggregates (RA) not only provides the solution for its deposit in landfills but also the preservation of natural resources resulting from the extraction of natural aggregates. The global average worldwide waste generation in construction and demolition was equivalent to 1.68 kg/capita/day in 2018.

Self-compacting concrete (SCC) is used to facilitate proper filling and structural performance of constrained and/or reinforced areas [9,10]. This material possesses different characteristics than traditional concrete, for example, in terms of strength. The idea of this material was first introduced in 1992 by Okamura [11] and has gained space over time in the construction industry [10,12], due to the ease of placement in hard-to-reach areas with less effort and time [13] The advantages of this type of material include technological, social and economic advantages; however, its cost is 2 to 3 times higher than that of conventional concrete due to the high demand for cementation materials and chemical admixtures (super plasticizers or water reducers).

A decrease in the carbon footprint for concrete could be achieved, effectively reducing carbon dioxide (CO_2_) emissions [14]. The use of RA in concrete leads to a 20% decrease in CO2 emissions approximately and preservation of natural resource by aggregate extraction as 60% [15]. A good part of global industries are working to be more sustainable, such as the construction sector [16]. Local and state administrations, the scientific community, and the general public are increasingly aware of the depletion of natural resources and their deterioration for sustainable development. As a result of this development, the need to recycle waste arises, in which resources become part of the circular economy and are preserved from generation to generation [17,18]. Society as a whole has realized the need to combine economic development with sustainability and environmental protection. In recent years, some studies on the use of these wastes with concrete have been published, however, they are minimal. Most of the studies use recycled coarse aggregates in the manufacture of concrete [19,20,21,22], the least use recycled fine aggregates [23,24,25,26], other studies use aggregates of different nature [27,28,29,30], although most are aggregates from concrete waste [31,32,33,34]. Other studies focus on the manufacture of mortars [35,36,37,38].

In this context, the European Strategy establishes the following objectives for waste policy in all its member states: (a) reduction of waste generated; (b) increase in recycling and reuse; (c) limitation of incineration; (d) limitation of the use of landfills [39]. With these premises, the need for waste management policies that reduce environmental and health impacts and improve the efficiency of available resources is clear. The long-term goal is to turn the world into a recycling society, avoiding waste and using waste as a resource wherever possible. The goal is to achieve much higher levels of recycling and minimize the extraction of additional natural resources. From this it can be understood that nowadays people across the globe are aware of reusing waste by recycling it. This has a significant impact not only on the environmental side, but also through using this waste, the cost of concrete itself is reduced apart from enhancing the concrete properties.

There are so many parameters in determining the SCC properties, in which the influenced parameter is design parameters [40]. Most of the properties of concretes are determined by the proper proportion of ingredients used in the mix. If there is a slight variation in the proportion of ingredients used, there is a drastic variation in fresh and hardened properties being reported in literature [40]. Some of the proportion commonly used by researchers is water to cement ratio, water to binder ratio, coarse aggregate to fine aggregate ratio, total aggregate to cement ratio, water to solid ratio, etc., [41]. Apart from these proportions, several ingredients directly influence the properties of concrete also reported in the literature [42]. These proportions and percentage or mass of ingredients used for the mix are known as design parameters [40]. Without considering mixing conditions and environmental conditions, these design parameters have a greater impact on concrete properties.

Due to the ease of casting and testing in actual site requirements, most construction sites are casting cubical or cylindrical specimens to determine the compressive strength and split tensile strength [43]. Concrete is good in compression and most of the hardened properties (elasticity and durability properties) are related to compressive strength. This is for ease of calculation of other properties through knowing the compressive strength. Concrete is weak in tension but when it is used as a flexural member, some part of concrete is subjected to tension. Hence, it is necessary to study the concrete properties concerning tension; one such experiment accepted widely by researchers and academicians is split tensile strength [44]. Irrespective of the size of specimens, these two geometrical shapes are accepted widely. These strengths are determined by researchers at various ages of curing period or various curing conditions but the most acceptable method of curing is normal curing condition, and the curing period is 28 days. Because at actual practice, 28 days curing will obtain almost 90% of strength at normal curing condition [45]. For simplification purposes, some studies in the literature use the strength ratio to determine other properties which may be either mechanical or durability properties [44]. The ultimate strain value in uniaxial tension is expressed in terms of the strength ratio [46]. The material constants defining the failure envelope are related to the strength ratio [47]. Hence, it becomes important to identify the effect of strength ratio on various grades of concrete with variation in proportion, as is also reported in the literature [44].

By utilizing waste material from the construction sector, without harming the environment (usage of natural aggregates) in larger proportions, natural aggregates should be replaced by RA. However, some international standards allow usage of RA in SCC up-to a certain limit and they more often discuss fresh concrete properties only. Most hardened properties of concrete depend on strength (compressive and split tensile strength) properties at any age. These mechanical properties mostly depend on the design parameters as discussed earlier [40]. Many researchers have reviewed SCC with recycled aggregate, which is available, but have not studied the effect of the design parameters (more than three parameters) on two strength parameters simultaneously. As of the author´s knowledge, this review based on different grades of concrete and on the ingredient’s proportions is the first of its kind. Hence, this review is based on the impact of the design parameters on the strength ratio of SCC with RA.

## 2. Review Methodology

### 2.1. Search Strategies

**Figure 1:**
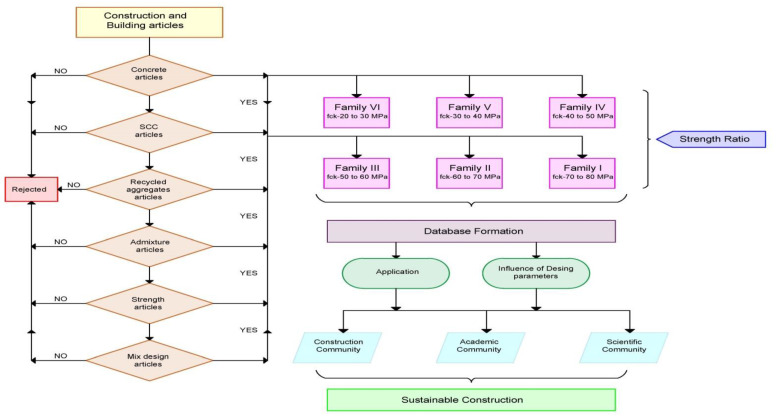
Review methodology.

The review methodology used in this review is reported in Figure 1. Reports on ingredients and mechanical properties of SCC from recent literature were searched and all other articles were omitted. More focus was given to SCC with recycled aggregates (both RCA and RFA), and were selected for further processes. Admixtures are added to SCC with several benefits like reducing the cost of the mix, enhancing the fresh and hardened properties, and increasing the homogeneity of the mix. Therefore, SCC with recycled articles and admixtures (mineral and chemical—both) were considered important for selecting articles. The design parameters were derived from the ingredients used in the mix for a given volume. Articles with both mechanical properties and design parameters were selected for this important review.

### 2.2. Data Extraction

From available literature on SCC with recycled aggregate, the design parameters were estimated using mix design (based on various methods). The collected literature should contain the SCC mix design as various ingredient weights in a given volume to satisfy the SCC properties (fresh and hardened). Since most of the literature did not concentrate on the powder particle in RFA or the natural fine aggregate, it was neglected for this study. Binder content included the cement and mineral admixtures i.e., binder material to bind aggregates together. W/C ratio, W/B ratio, TA/C ratio, FA/CA ratio, SP (kg/m^3^), W/S ratio (%), % of RFA, % of RCA, the fresh density of mix, compressive load area and split tensile strength load area were different design parameters considered for the current study. Total aggregates are the sum of fine aggregate, coarse aggregate and recycled aggregates, from which is given total aggregates to cement ratio. Overall solid contents in the mix like aggregates, recycled aggregates, cement and binders from which the water to solid ratio were estimated. The articles without mixed proportions, or any one of the required ingredients were omitted from the review process.

Based upon compressive strength grade of SCC with recycled aggregate, six families were divided. The parameters like the different international codes or standards for specimen testing, the shape of the specimen, curing condition of specimens, and size of specimens were not taken into account, while considering the 28 days compressive strength of control or reference SCC mix. Initially, the families were classified based on the control or reference SCC mix and 28 days compressive strength. The 28 days compressive strength for control or reference mix lies in the range of 70 MPa to 80 MPa, grouped as a family I. Similarly, family II, family III, family IV, family V and family VI consisting of 28 days compressive strength and lie in the range of 60 MPa to 70 MPa, 50 MPa to 60 MPa, 40 MPa to 50 MPa, 30 MPa to 40 MPa and 20 MPa to 30 MPa. With respect to compressive strength in each family, the corresponding split tensile strength is also tabulated. Concerning compressive strength, the parameters like international codes or standards for specimen testing, shape of the specimen, curing condition of specimens and size of specimens are not considered. In absence of any one of the strengths at 28 days, the article itself is omitted for the review purpose.

## 3. Design Parameters from Literature

The family I consist of three articles which consist of control mix and compressive strength lying between 70 MPa to 80 MPa—their values are tabulated in Table 1 along with split tensile strength in the range of 2.50 MPa to 4.46 MPa. Gesoglu et al., 2015 [48] used nine different mix proportions as shown in Table 1. The lower the W/C or W/B ratio, the higher the compressive strength is observed from family I. TA/C ratio lies in the range of 3.3 to 5.2 for high strength mix. FA/CA ratio lies in the range of 1.7 to 2.5 and SP available in the mix is in the range of 2 kg to 7 kg. Replacement of natural aggregates with 100% of recycled aggregates results in higher compressive strength. The lower the W/S ratio, the higher the compressive strength, the higher the W/S ratio, the lower the compressive strength, as observed from Table 1. The highest W/S ratio is observed from Sadeghi-Nik., et al. 2019 [49]. W/S ratio is in the range of 5.1% to 8.4% is observed for a higher strength mix. Gesoglu et al. 2015. [48], observed that although there was higher compressive strength, there was no corresponding increase in split tensile strength.

For family II, there are four articles with compressive strength at 28 days for control or reference SCC in the range of 60 MPa to 70 MPa, as detected in literature. Compared to Table 1, the compressive strength for family II in Table 2 is decreased due to an increase in W/C and W/B ratio. For family II, the TA/C ratio lies in the range of 4.0 to 5.7 and increased compared to the family I. FA/CA ratio for family II is in the range of 2.1 to 3.8 which is also increased compared to family I. SP used for family II is in the range of 1.8 to 6.6 which is decreased compared to the family I. Compared to family I, the W/S ratio for family II lies in the range of 3.4% to 8.1%. The literature considered for family II has 100% replacement for their natural aggregates by recycled aggregates. Family II consists of split tensile strength at 28 days in the range of 2.20 MPa to 5.30 Mpa.

For family III, the reference or control SCC compressive strength at 28 days is in the range of 50 MPa to 60 MPa and the design parameters are tabulated in Table 3. Ten articles with control SCC compressive strength in the range of 50 MPa to 60 MPa along with split tensile strength in the range of 0.96 MPa to 5.50 are tabulated in Table 3. Compared to families I and II, an increase in W/C and W/B ratio is observed which results in a decrease in compressive strength. FA/CA ratio lies in the range of 1.5 to 6.1 for family III, which is higher than family I and II. Higher TA/C ratio results in a higher W/C ratio as reported by Aslani et al., 2018 [54] and Guo et al., 2020 [55], effectively leading to a higher W/S ratio in percentage. Aslani et al., 2018 [54] also used a larger proportion of binder material in the mix to increase in SP content and W/B ratio. Apart from research by Aslani et al., 2018 [54] there is shown to be some relationship between design parameters and strength. Increase in W/S ratio when compared to Family I and II are due to more quantity of available liquid in the system.

Family IV consists of ten articles from the literature, with reference mix compressive strength at 28 days in the range of 40 MPa to 50 MPa and their corresponding split tensile strength in the range of 1.80 MPa to 5.17 MPa, along with design parameters as tabulated in Table 4. Due to a decrease in compressive strength for family IV, there is a decrease in W/C and W/B ratio for Family I, Family II and Family III observed. Except for Kou et al., 2009, there is a decrease in usage of SP when compared to Family I, II and III. An increase in W/C or W/C ratio counteracts a decrease in usage of SP. An increase in W/S when associated with other previous families is due to the availability of more water in the system which results in a decrease in strength. TA/C lies in the range of 3.3 to 7.8 and FA/CA lies in the range of 1.5 to 3.6, when compared to other families, these two ratios are decreased. Manzi et al., 2017 [62] and Nili et al., 2019 [63], replaced natural fine aggregate with recycled fine aggregate and simultaneously natural coarse aggregate with recycled coarse aggregate.

Family V consists of eight articles from literature with reference SCC compressive strength at 28 days lying in the range of 30 MPa to 40 MPa and corresponding split tensile strength lying in the range of 2.28 MPa to 4.12 MPa at 28 days as tabulated in Table 5. Apart from Aslani et al., 2018 [54] and Babalola et al., 2020 [67], there are no drastic increases shown in W/C or W/B ratio when compared to other literature in Family V. Compared to previous families, there is an increase in W/C and W/B ratio observed for family V. Requirement of SP is reduced to 1.90 kg to 6.10 kg for family V when compared to other families due to increase in water content of the mix. Due to an increase in water content, there is an increase in the W/S ratio for family V observed. An increase in TA/C and FA/CA ratio is observed for family V, when compared to all other families. Bahrami et al., 2020 [12], Sun et al., 2020 [68] and Surendar et al., 2021 [69] used a constant TA/C ratio, FA/CA ratio and W/S ratio in percentage and reported a slight variation in strength requirement for minimum modification in aggregates. Several authors tried 100% replacement for natural aggregate and achieved the minimum requirement of strength.

Three articles from literature consist of reference mix compressive strength of SCC in the range of 20 MPa to 30 MPa, constituent Family VI and their corresponding split tensile strength in the range of 1.61 MPa to 3.07 MPa, along with design parameters as tabulated in Table 6. W/C and W/B ratio is found to be high among families for family VI result in lower strength properties. Lowest TA/C and FA/CA are found to be high among the families, which results in higher water requirements for this family. Due to increasing the fresh concrete properties, there is an increase in SP content observed for family VI. Due to the increase in water content and SP content for family VI, there is an increase in the W/S ratio observed for family VI.

### Relationship between Compressive Strength and Split Tensile Strength to Compressive Strength Ratio for Different SCC Grades

**Figure 2:**
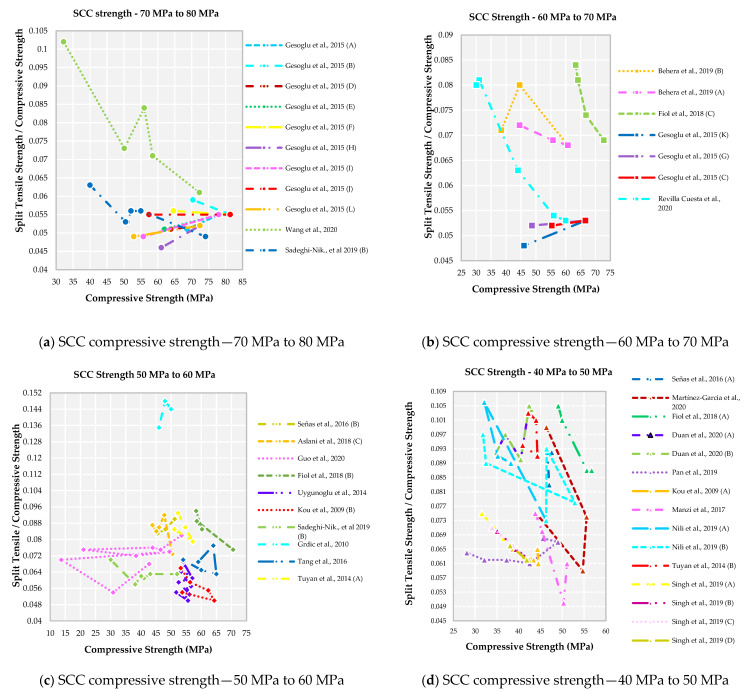

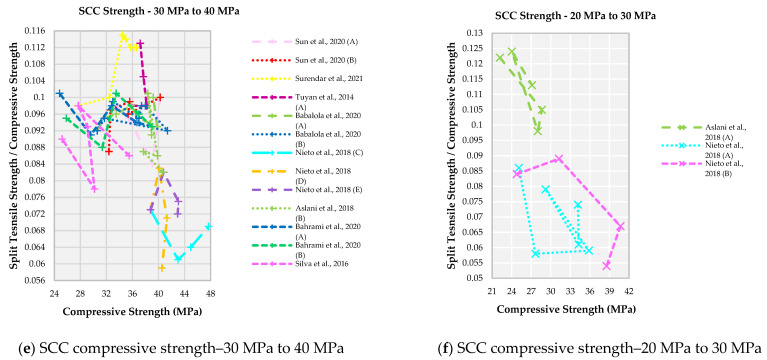
Relationship between ratio of split tensile strength to compressive strength and compressive strength according to literature for SCC recycled aggregate concrete.

The relationship between the ratio of split tensile strength to compressive strength and compressive strength of SCC recycled concrete for various strength grades are shown in Figure 2. It is also observed that concerning different concrete compressive strength grades, there is not an identical relationship between strength ratio and compressive strength. A higher split tensile strength for a medium-strength grade is observed when compared to the high and low strength grade of SCC with recycled aggregate.

For the family I, Wang et al., 2020 [50] observed the split tensile strength to compressive strength ratio as highest in the range of 0.065 to 0.1, as observed from Figure 2a. Among the family I, Wang et al., 2020 [50] possess higher split tensile strength, which results in a higher strength ratio. The remaining authors have a strength ratio in the range of 0.045 to 0.065 due to medium split tensile strength. Except for Wang et al., 2020 [50] and Sadeghi-Nik et al., 2019 [49], the strength ratio decreases with an increase in compressive strength observed whereas for the remaining authors the strength ratio increases with increases in compressive strength. The strength ratio converges at a point of 0.055, when compressive strength greater than 75 MPa observed.

With the decrease in compressive strength grade for family II, there is a decrease in strength ratio observed from Figure 2b. Fiol et al., 2018 [52] observed that there is a drop in ratio with an increase in compressive strength. Out of seven works of literature, results from four works show that there is an increase in strength ratio with a decrease in compressive strength, and the remaining three works show vice versa results. The range of strength ratio lies between 0.048 to 0.084 for compressive strength of range 73 MPa to 30 MPa.

The family III, strength ratio majorly lies between the ranges of 0.050 to 0.093 for compressive strength of range 71 MPa to 14 MPa observed from Figure 2c except for the Grdic et al., 2010 [59] which shows a higher strength ratio. Grdic et al., 2010 [59], has higher split tensile strength and medium compressive strength which results in a higher strength ratio. Most of the authors observed that with a decrease in compressive strength there is a decrease in strength ratio observed. Aslani et al., 2018 [54], Uygunoglu et al., 2014 [57] and Tang et al., 2016 [60] had higher split tensile strength than control or reference mix for their first replacement of natural aggregate by recycled aggregate which resulted in an increase in strength ratio compared to other literature.

For the family IV, the strength ratio lies between the range of 0.049 to 0.105 with compressive strength lying in the range of 57 MPa to 27 MPa as observed from Figure 2d. It is observed that with a decrease in compressive strength, the strength ratio slowly increases because of the lower compressive strength value with the same split tensile strength value. Nili et al., 2019 [63] and Singh et al., 2014 [1] show the higher split tensile strength than the control mix, resulting in a higher strength ratio. Even though there was a sudden drop in the compressive strength found in certain literature, there is no such drop of strength observed for split tensile strength.

The strength ratio lies in the range of 0.059 to 0.115 with compressive strength lying in the range of 25 MPa to 48 MPa for the family V, as observed from Figure 2e. A unique feature of assembling strength ratio in the range of 0.092 to 0.1 is observed in this family when compared to all others. Several articles of literature with strength ratio increases with a decrease in compressive strength and the vice-versa cases are similar in the literature observed. Surendar et al., 2021 [69] show a higher strength ratio without any admixtures resulting in lower usage of SP.

For family VI, the strength ratio lies in the range of 0.054 to 0.122 with compressive strength lying in the range of 22 MPa to 42 MPa as observed in Figure 2f. Aslani et al., 2018 [54] show a higher strength ratio due to the presence of three admixtures, resulting in densely packed materials contribute to higher strength. Aslani et al., 2018., did not replace the natural aggregate with recycled aggregate more than 50% which resulted in higher strength values. The remaining articles show a strength ratio lying in the range of 0.054 to 0.089.

In general, the strength ratio decreases with increasing compressive strength (irrespective of the grade of concrete) at a decreasing rate as observed and already reported in literature [44]. It can be explained as: the increasing rate of split tensile strength occurs at a much smaller proportion compared to the increasing rate of compressive strength. These results are in agreement with findings in the literature [44]. It is also observed that the strength ratio is 0.050 to 0.120 for the lower grade of SCC [Family V and VI] and increased to the range of 0.050 to 0.150 for a medium grade of SCC [Family III and IV]. For a higher grade of SCC [Family I and II], the strength ratio is decreased in the range of 0.045 to 0.110. These findings agree with those results observed in literature [44].

## 4. Impact of Design Parameters on Strength Ratio

### 4.1. Impact of W/C Ratio on Strength Ratio

**Figure 3:**
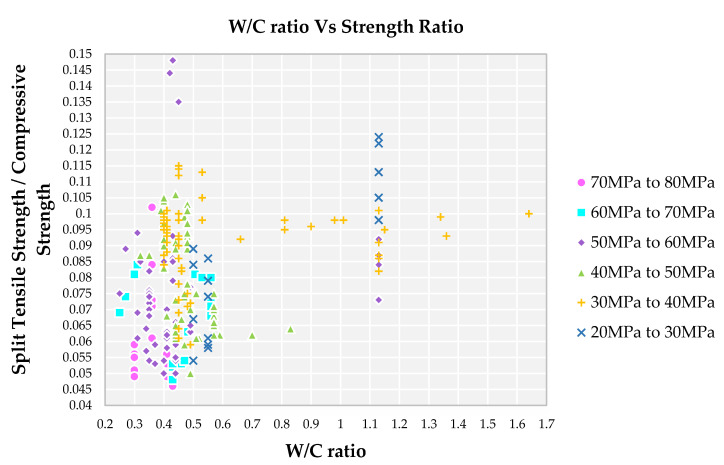
Impact of W/C ratio on split tensile strength to compressive strength ratio for different grades SCC concrete with recycled aggregate according to literature.

The strength ratio initially increases with an increase in W/C ratio as observed in Figure 3 up-to a W.C ratio of 0.45 and after that with an increase in W/C ratio, resulting in stabilization of strength ratio. This stabilization of strength ratio is due to the particle packing of ingredients (presence of more amounts of powder content) used for a mix which gives minimum strength even given more water. One of the prime characteristics of SCC, whenever we are checking for mechanical properties, is that it should first pass-through fresh properties. If it has a less or inadequate amount of water, the aggregates are segregated outside resulting in poor mechanical properties [71]. If there is more water, then there is a chance of bleeding and segregation of aggregates which affects the strength properties of SCC itself [72]. Hence, the optimum amount of water should be maintained in the mix to get the proper fresh and hardened properties of SCC.

Initially the decrease in strength ratio is due to lower split tensile strength and higher compressive strength at 28 days. A lower W/C ratio results in the lesser formation of hydration products, the particle packing playing a key role in increasing density, thereby increasing compressive strength and split tensile strength. On further increasing the W/C ratio, compressive strength is decreased at a higher rate when compared to split tensile strength. Therefore, the strength ratio goes higher for the lower grade of SCC for a W/C ratio of 0.40 to 0.60. Water available in the cement matrix is the adequate amount for the W/C ratio lying between 0.30 to 0.45 to form hydration compounds which make the cement paste matrix denser and bind the aggregates [73]. Due to the presence of recycled aggregates, there is an increase in the W/C ratio, which results in drop in strength characteristics. Recycled aggregate consists of old mortar adhered to the surface, which is porous in nature, absorbing the additional water to maintain the same workability nature of SCC [40]. And also due to the irregular texture and shape of recycled aggregate, additional water is required to make a suspension in cement mortar pasteThis excess water will evaporate during the hardening process and make the cement paste matrix as porous which leads to a drop in strength characteristics [73], which leads to a drop in strength characteristics. Presence of old mortar from RA in cement paste matrix are porous in nature, which absorbs water and it makes even weaker [40].

### 4.2. Impact of W/B Ratio on Strength Ratio

To achieve minimum fresh properties, there is a need for a substantial amount of fine particles in the concrete. However, to obtain the minimum requirement for hardened properties there is no compromise in the aggregate content in SCC. If we increase the cement content in the mix, the cost of the mix increases and other problems are created alongside high energy consumption and carbon dioxide emissions. Without compromising the minimum quality with respect to both fresh and hardened properties of SCC, the increase in fine particles is achieved by the addition of admixtures. Admixture not only reduces the cost of the mix but also enhances the concrete properties and its environmental protection [74]. Mineral admixtures are used to reduce the overall chemical admixtures and cement required and also provide additional advantages like reducing the internal friction and viscosity during fresh state [75]. Reduction in cement content results in a reduction in the amount of heat generated during hydration and enhances resistance to thermal cracking [75]. Apart from the reactive part of mineral admixtures, the unreactive part will fill the voids in cement mortar paste to make the SCC; impervious to chemicals, water and with a restricted expansion of alkali aggregates [75].

**Figure 4:**
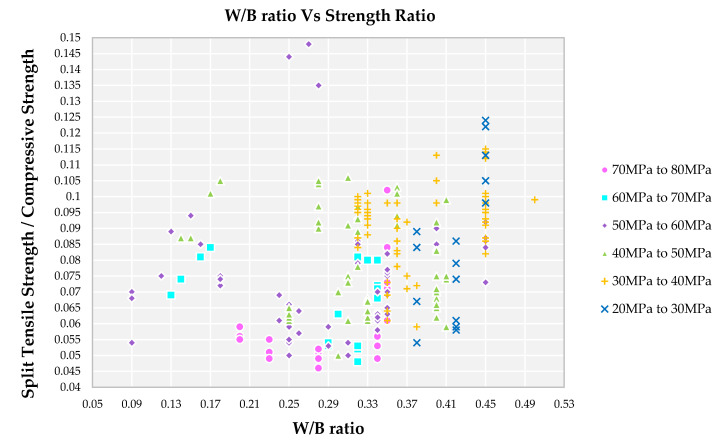
Impact of W/B ratio on split tensile strength to compressive strength ratio for different grades of SCC concrete with recycled aggregate according to literature.

In literature, various types of admixtures like fly ash, lime stone, ground granular blast furnace slag, etc., are used as a mineral admixture for the replacement of cement content [54,66,68]. Some of the admixtures possess pozzolanic properties and others are used as filler material in SCC [66]. Another advantage of using mineral admixture is to counteract the adverse effect caused by the recycled aggregates in SCC [76]. Ingredients that possess the nature of binding of other ingredients together are termed as a binder which includes cement and mineral admixtures. During hydration reaction, an additional compound formed—calcium hydroxid—which on hardening process evaporates and forms pores. In the presence of mineral admixture, the silica from it reacts with calcium hydroxide and forms extra calcium silicate hydrate, thereby reducing the pore size further. Furthermore, the calcium oxide from mineral admixture reacts with silica present in sand or binder to form additional calcium silicate hydrate thereby enhancing the properties [76]. If the additional ions are not available, the admixture fills these small pores without reacting thereby increasing the density. This reason for the lower W/B ratio having a higher grade of compressive strength of SCC is observed from Figure 4.

### 4.3. Impact of TA/C Ratio on Strength Ratio

Total aggregate plays a vital role in determining the dimensional stability apart from fresh and hardened properties of concrete, which occupies 65% to 70% in total volume [51]. The ingredients of the concrete mix are packed properly only when the distribution of total aggregates in a given volume is properly maintained. Hence, to get proper packing of the total volume of aggregates, the proper particle size distribution of aggregates in the given volume must be maintained. To distribute evenly in a given volume, the amount of paste required to distribute it plays a vital role. Hence, to achieve the maximum density of concrete, the TA/C ratio should be maintained in an optimum manner. Cement mortar paste content should also be maintained in an optimum manner, in such a way that total aggregates are suspended in it. Poor segregation resistance results in poor deformability, non-homogeneous nature of hardened concrete and obstruction around congested reinforcement [77]. Hence, the segregation resistance plays a significant role for SCC and to achieve higher segregation resistance, the powder content should be increased to an optimum level.

**Figure 5:**
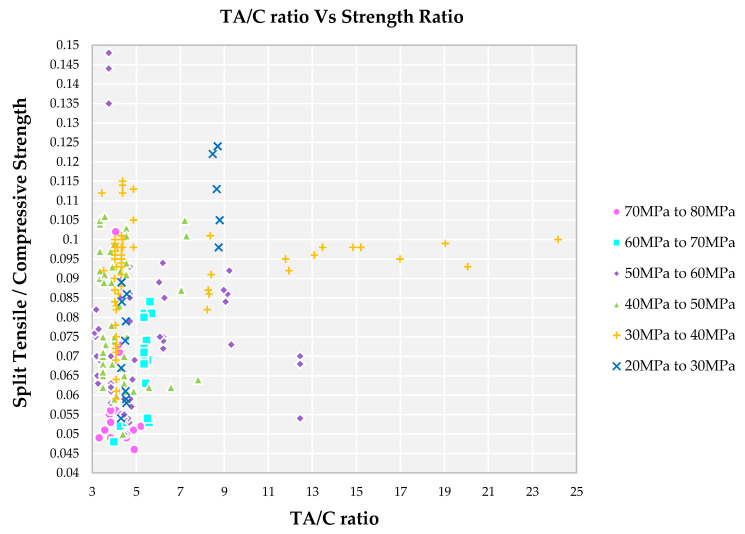
Impact of TA/C ratio on split tensile strength to compressive strength ratio for different grades of SCC concrete with recycled aggregate according to literature.

A lower grade of concrete results in a higher strength ratio because the change in compressive strength is at a higher level when compared to the change in split tensile strength. For the lower TA/C ratio, a higher strength ratio is observed from Figure 5 for family V and VI. This indicates that the cement paste formation is more to bind the coarse aggregate, which results in more pores at a hardened state. At a TA/C ratio greater than 3.0, the strength ratio becomes higher for the family V and VI. This indicates that the cement paste content is less and aggregates content becomes more, resulting in more voids in the cement matrix. A higher grade of concrete results in a lower strength ratio because the small change in split tensile strength is divided by a higher change in compressive strength. A strength ratio of 0.045 to 0.06 for a higher grade of concrete (Family I and II) with a lower TA/C ratio is observed in Figure 5. A lower TA/C ratio between 1.5 and 3.0 has a higher strength ratio for the family I, II, III and IV because adequate mortar paste is formed in which coarse aggregates are initially wetted and float in the fresh state. At a hardened state, the paste becomes denser and the formation of ITZ around coarse aggregate with minimum pores yields higher compressive strength.

### 4.4. Impact of FA/CA Ratio on Strength Ratio

The fresh concrete state of SCC requires the appropriate proportion of coarse aggregate and particle size distribution within the specified limit to promote the movement of ingredients and the filling of the voids in between them [75]. The rheological characteristics of the cement mortar matrix play a vital role in determining the flowability and segregation resistance of SCC in the fresh state, which may be correlated with the FA/CA ratio. The lower the FA/CA ratio, there is poor flowability and a high obstruction tendency of SCC [78]. When FA/CA ratio is higher than 4.0, there is an excessive amount of SP required to obtain fresh concrete properties [78]. An increase in FA/CA ratio results in a decrease in strength. This is due to an increase in the FA content which increases the specific surface area of the aggregates, fine particles and very fine particles [78]. This will lead to an increase in water content which is required to wet the surface of aggregates, fine particles, and very fine particles [78]. Water demand will increase further to form the required amount of cement paste to coat all the FA [78]. An increase in the replacement of fine aggregates by coarse aggregates leads to an increase in the FA/CA ratio, resulting in a decrease in compressive strength [75]. A higher FA/CA ratio shows the smaller gap between aggregates resulting in a squeeze out of paste and thus a lesser amount of hydration product formation.

**Figure 6:**
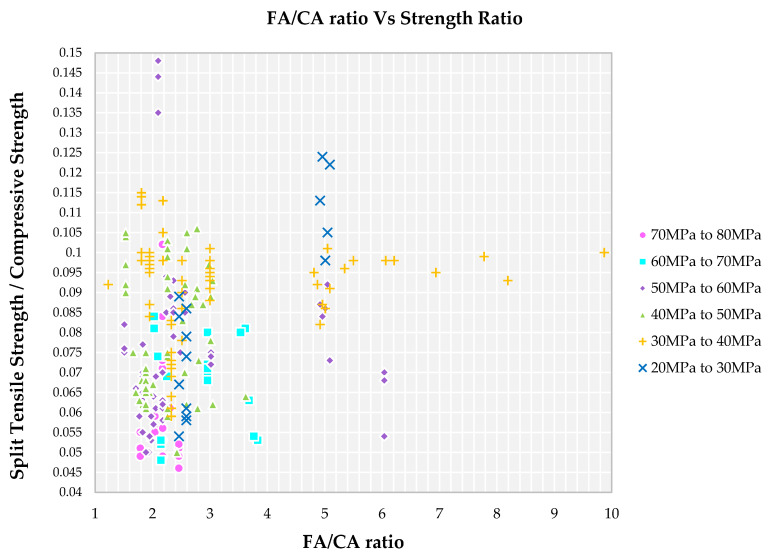
Impact of FA/CA ratio on split tensile strength to compressive strength ratio for different grades of SCC concrete with recycled aggregate according to literature.

Family I, II, III and IV show a FA/CA ratio in the range of 1.5 to 2.5 from Figure 6 for a lower strength ratio. Fine aggregate present in the cement matrix not only fills the voids or pores but also provides the binding capacity in the hardened state. However, for the same FA/CA ratio in the range of 1.5 to 2.5, the strength ratio is raised and there is a drop in compressive strength of SCC for family V and VI observed. This indicates that apart from FA/CA ratio, other factors also influence the strength ratio of SCC. A higher level of FA/CA ratio, i.e., greater than 3.0, is observed for the family IV, V and VI from Figure 6. The presence of a larger amount of fine aggregate indicates that there is not enough quantity of binder to bind it to form a paste which results in pores around it. The trend of reducing strength with increasing fine aggregate content may be attributed to crack tortuosity because increased fine aggregate content will lead to a shorter path that a crack needs to follow to go from one side of a sample to another. This is the reason for the higher FA/CA ratio for the families IV, V and VI. And for the family I, II and III, the greater the amount of coarse aggregate, and so greater tortuosity, will mean higher energy required to propagate the crack, and so higher strength. And also, the higher the FA/CA ratio, the higher the specific area, the paste content available is enough to coat the aggregates, thereby leaving reduction paste formation for lubrication requirements, which resulted in small gaps in between aggregates [75].

### 4.5. Impact of TA/C Ratio on Strength Ratio

The governing factor for fresh concrete properties of SCC is ingredients used in the mixes and their proportion. An appropriate proportion of ingredients, specifically particle size distribution of aggregates to fill the voids in between them by use of both aggregates and paste [75]. The total aggregates (TA) include the fine and coarse aggregate used in the mix including their different sizes. Some main properties of ingredients like shape, size, fineness modulus, grading limit, specific gravity, water absorption, etc., had much impact on the resulting concrete properties at hardened state [75]. SCC makes use of mineral admixtures, increasing the specific gravity and consistency of cement mortar paste, which in turn provides enough suspension to prevent the segregation of aggregates [75]. If there is enough quantity of paste formation, every grain in fine aggregate is coated and balance paste is used for lubrication of coarse aggregates. Hence, the TA/C ratio plays a vital role in determining the properties of concrete both in the fresh and hardened state. The higher TA/C ratio produces larger gaps between fine aggregates and coarse aggregates, leaving more space for paste resulting in a reduction in strength.

**Figure 7:**
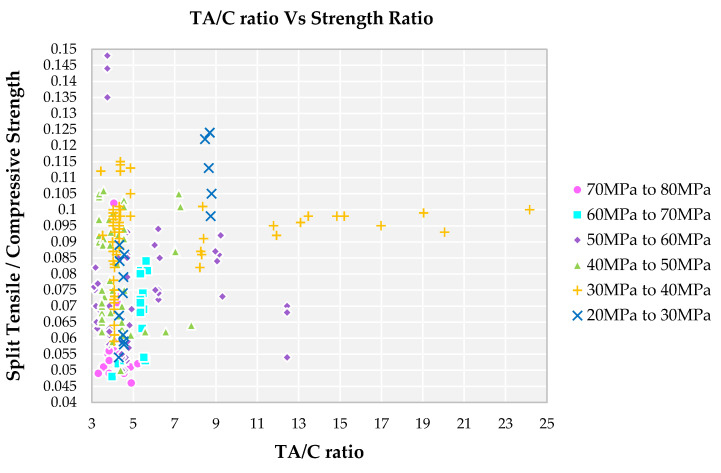
Impact of TA/C ratio on split tensile strength to compressive strength ratio for different grades of SCC concrete with recycled aggregate according to literature.

The strength ratio for family I and II is lowest among all the families and it is in the range of 0.045 to 0.05 for TA/C ratio, lying in the range of 3 to 5 from Figure 7. Higher compressive strength with medium split tensile strength results in a lesser ratio. The optimum quantity of paste results in complete wet of fine aggregate leads to suspension of coarse aggregate leads to increase in strength [75]. The minimum strength ratio for families III and IV is 0.055 with TA/C in the ratio of 3 to 9. Family V and VI has the highest strength ratio with the TA/C ratio in the range of 5 to 23. Presence of a larger amount of total aggregate in the mix results in more pores in the cement matrix. Higher range of TA/C ratio results in minimum amount of paste to bind the aggregates and results in formation of cracks whereas the families with lower TA/C ratio, there is insufficient formation of hydration product results in improper binding of aggregates.

### 4.6. Impact of Superplasticizer Weight on Strength Ratio

Particle friction between coarse aggregate, fine aggregate and binder content increases the internal resistance to flow, hence limiting the deformability and speed of flow. Chemical admixtures dissolve through the cementation of particles through surface activating action among solid and liquid states, reducing the water quantity. Few groups of chemical admixtures offer the negative potential to cement and cementation particles; thus, the binding particles become electrostatically repelled from each other and fluidize the cement mortar paste [79]. To improve the fresh and hardened concrete properties, the addition of mineral and chemical admixtures is necessary to counteract the negative impact caused by recycled aggregates [58]. In most cases, SCC gains more advantages than conventional concrete in terms of workability, mechanical, structural compactness and durability; however, the performance results depend heavily on the chemical admixture [80]. SP dry extract content in the SCC directly affects their mechanical behavior due to the adsorption phenomenon of SP (electrostatic and steric repulsion) as a sign of changes in the cement hydration kinetics [81,82]. The SP with the largest dry extract had a greater ability to prevent flocculation of cement particles, which generates an increase in the mechanical properties.

**Figure 8:**
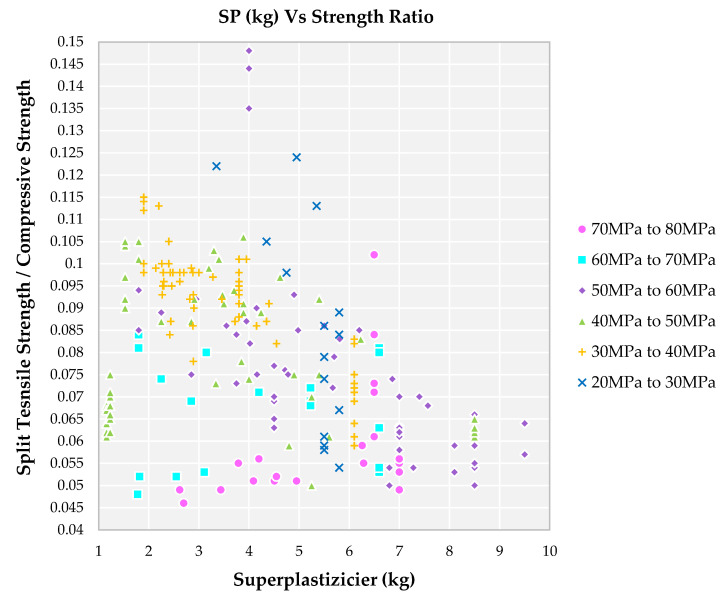
Impact of Superplasticizer weight on split tensile strength to compressive strength for different grades of SCC concrete with recycled aggregate according to literature.

Families I, II, III and IV have SP content up-to 9 kg in the mix resulting in a lower strength ratio in the range of 0.04 to 0.07, as observed from Figure 8. For a higher grade of SCC mix, the mineral admixtures used will be more hence the requirement of SP became more. Initially, at a lower dosage of SP, a lower segregation level is a result of higher cohesion and segregation resistance; therefore, the strength ratio is low for a higher grade of SCC. And on a further increase of SP, the water between cement particles releases, and an increase in the water films coats the mixture particles [83,84]. Hence, the fluidity of the mix increases and results in the packing of ingredients in a more prominent manner which contributes to an increase in density. Families V and VI have a higher strength ratio with SP content in the range of 2.0 to 6.0 kg. Higher SP content lowers the strength ratio due to delayed mixing and casting time which reduces the self-compacting ability of the concrete, thereby increasing its confined air bubbles [85].

The fluidity and packing characteristics of SCC depend on the W/S ratio of the mix, because the solid content of the mix is suspended in the fluid system i.e., water and SP. Coarser ingredients like coarse aggregate are one type of solid along with finer ingredients like fine aggregates and binder content. These finer ingredients should be in proper proportions to form the paste with appropriate viscosity and flowability. The minimum water content present in the cement matrix is used for hydration reaction along with wetting the finer ingredients’ surface completely so that it fills voids [86]. Cement mortar paste content should be at an optimum level so that coarse aggregate is suspended in this fluid system. Hence, it is more important to maintain this W/S ratio at a proper level as well as lubricant behavior.

**Figure 9:**
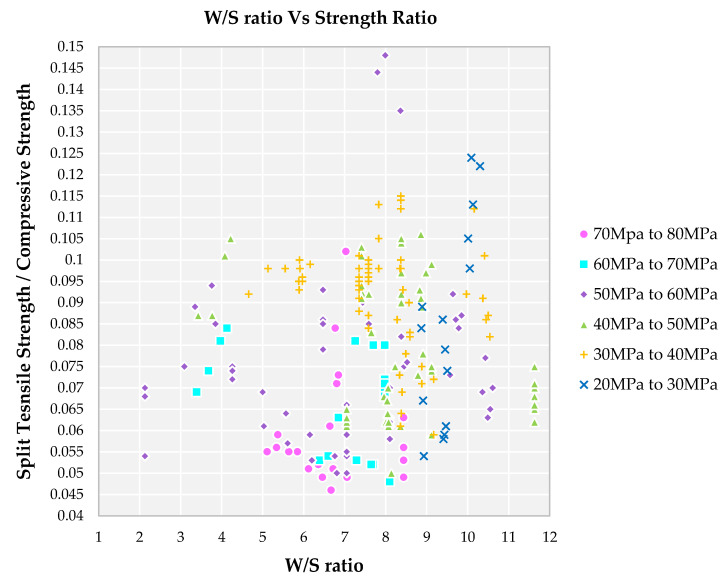
Impact of W/S ratio on split tensile strength to compressive strength for different grades of SCC concrete with recycled aggregate according to literature.

A lower W/S ratio results for the families II and III but strength ratio is in the range of 0.065 to 0.095 from Figure 9. Higher compressive strength of families is observed due to the water content available being optimum for lubrication and hydration purposes, which will have a denser cement mortar matrix to bind the aggregates. A higher W/S ratio results in a higher strength ratio for the families V and VI from Figure 9. A higher W/S ratio results in higher water content in the cement matrix at the fresh state and at hardened state this water evaporation leads to porosity. More pores result in a decrease in mechanical properties of the SCC mix.

### 4.7. Impact of Percentage of RFA on Strength Ratio

RFA contains finer hydrated or non-hydrated particles that form a weak link in new hydration product formation. This leads to a decrease in the density of the cement matrix. Loss in strength is due to an increase in drying shrinkage which is due to more amount of paste volume in the mix [87]. The presence of more paste volume is due to the old paste present in RFA and the new paste required to bind the aggregates [88]. Most authors in the literature suggest that special attention should be given to the case of 50% replacement of NFA by RFA to achieve RFA blended SCC strength nearer to that of reference or control SCC [85]. For a higher level of replacement of NFA, RFA properties should be enhanced [88]. The presence of RFA in SCC causes an increase in the average pore size at younger ages; however, for older ages, there is a decrease in the average pore size which leads to a denser cement matrix and contributes to the mechanical properties [85]. Furthermore, the smaller particles of RFA fill these smaller pores leading to a filler effect thereby increasing the mechanical properties of RFA blended concrete [58]. Salesa et al., 2017 [14] observed that mechanical properties were higher due to the presence of the higher amount of non-hydrated particles from recycled aggregates (Predominately RFA) which lead to hydration process and more formation of hydration products. The presence of mineral admixture as one of the ingredients leads to the pozzolanic reaction between the calcium hydroxide (product from hydration reaction) and pozzolanic minerals (silicon dioxide and aluminium dioxide), leading to the formation of additional calcium silicate hydrate which contributes to mechanical properties [58]. Apart from this additional CSH formation, RFA also possesses some self-sustaining binding materials which also contribute to mechanical properties [58].

**Figure 10:**
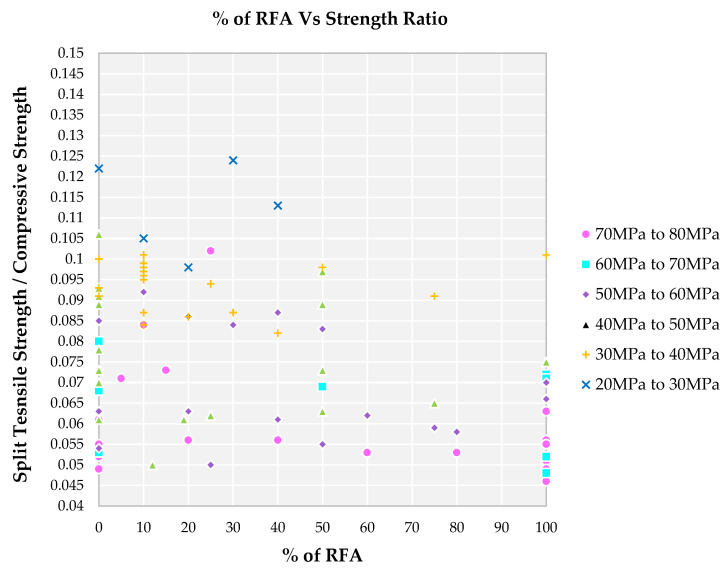
Impact of percentage of RFA on split tensile strength to compressive strength for different grades of SCC concrete with recycled aggregate according to literature.

From Figure 10, it is observed that the only minimum replacement percentage of NFA by RFA did not show any such difference in the strength ratio. The maximum replacement percentage for NFA by RFA for various grades of SCC is observed as 20%. Lower replacement of NFA by RFA results in a higher strength ratio and higher replacement of NFA by RFA results in a lower strength ratio as observed in family I. There is an increase in strength ratio for family II when compared to family I for a different level of replacement of NFA by RFA. When compared to the family I and II, family III possess a higher strength ratio in the range of 0.054 to 0.085 concerning a different level of replacement of NFA by RFA. A 100% replacement of NFA by RFA is observed for families I, II and III alone as observed from Figure 10. An increase in strength ratio for family III concerning all other families and a smaller number of studies are done for a higher level of replacement. Family IV, V and VI possess a lower level of replacement when compared to other families and fewer investigations leads to higher a level of strength ratio.

### 4.8. Impact of Percentage of RCA on Strength Ratio

The presence of more amounts of RCA content results in greater porosity and the larger gap between aggregate paste interface which could be due to the presence of debris attached to the surface of RCA [89]. Due to the production process of RCA, there is a chance of microcracks and damage to its surface [68]. The decline in the compressive strength of RCA blended concrete is due to the poor quality of RCA. Old mortar attached to the surface of RCA deteriorates the ITZ bonding strength and further reduces the mechanical properties [54]. The bonding strength of the new ITZ is much superior to that of the old ITZ and cracks easily, propagating through the old ITZ, consequently reducing the mechanical properties [68]. Natural aggregate resulted in lower bending resistance due to their smoother surfaces whereas RCA possesses higher bending resistance to increase mechanical properties [90]. Earlier strength development of RCA blended SCC has lower strength when compared to the later hardening age of SCC.

Most of the literature state that 20% replacement of NCA by RCA shows higher strength than control concrete, due to good adhesion between the new mortar and the adhered mortar of recycled aggregate as well as the quality of RCA [62]. Most of the studies in the literature suggested the 50% replacement of NCA by RCA for longer curing periods, the strength of RCA blended SCC reaches nearer to that of control or reference SCC [91]. Compared to RFA, RCA shows a greater negative effect due to the lower strength of RCA than NCA predominately [92]. The reduction in mechanical properties of RCA blended SCC is due to higher water absorption characteristics of RCA, leading to a higher W/C ratio [93]. A well known factor in concrete technology is that higher W/C ratio leads to the lower the mechancial properties of concrete. To achieve higher mechanical properties, the properties of RCA are enhanced and several authors achieved higher replacement levels [62]. Incorporation of RCA at a lower level does not affect the mechanical properties much, as reported by [62]. A clear trend for the effect of RCA on strength ratio has not been established in literature so far. It purely depends on the quality of RCA used apart from other ingredients in the mix.

**Figure 11:**
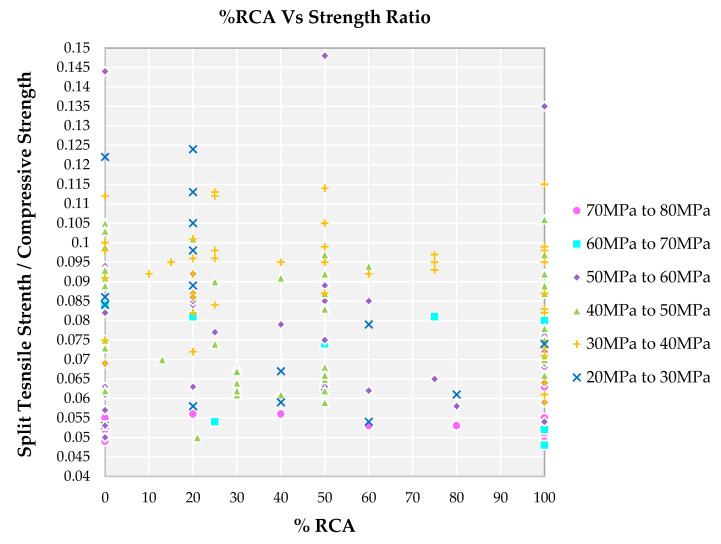
Impact of the percentage of RCA on various compressive strength of SCC concrete with recycled aggregate according to literature.

The strength ratio for all the families lies in the range of 0.05 to 0.115 for most of the cases, as observed in Figure 11. It is observed that 100% replacement of NCA by RCA is observed for most of the family I and the strength ratio lie in the range of lower order in the range of 0.046 to 0.070. Family II also had 100% replacement of NCA by RCA resulting in a slight increase of strength ratio in the range of 0.048 to 0.080. Different levels of substitution of NCA by RCA is found for family III, for higher substitution there is lower strength ratio and for lower substitution a higher strength ratio is observed. This is because if the order of compressive strength is higher and split tensile strength is lower the result is a higher strength ratio for families I, II and III. Even higher replacement of NCA by RCA results in a higher strength ratio as observed for family IV. Family V and VI possess a higher strength ratio for all the replacement levels when compared to all other families. This is because the decrease in compressive strength is much higher in order compared to that of a decrease in split tensile strength for family V and VI.

### 4.9. Impact of Fresh Density on Strength Ratio

The lower density of recycled aggregates and an excessive amount of paste leads to a weak interface zone and low-density matrix generating a poor matrix [62]. ITZ in recycled aggregate concretes showed that more voids and the presence of calcium hydroxide exist in both old and new paste formations in RAC [94]. The thickness of the older ITZ in the range of 40–50µm and for the newer ITZ is in the range of 55–65µm [94].

The microstructure of ITZ in concrete is extremely different from the ITZ found in cement paste due to longer portlandite crystals, higher porosity and ettringite content in it [94]. The microstructure of RCA shows more voids on its surface [89]. These characteristics are responsible for the higher water absorption and more porosity of SCC at hardened state which results in less dense microstructures [89]. This leads to weakened bonding of ITZ between the pastes and aggregates [89]. The presence of recycled aggregates in SCC results in a lower fresher density than natural aggregate SCC due to the lower density of recycled aggregates and higher water absorption characteristics of recycled aggregates [56]. Due to the lower density of RFA and RCA used as a replacement for NFA and NCA, the density of RFA and RCA blended SCC is reduced.

**Figure 12:**
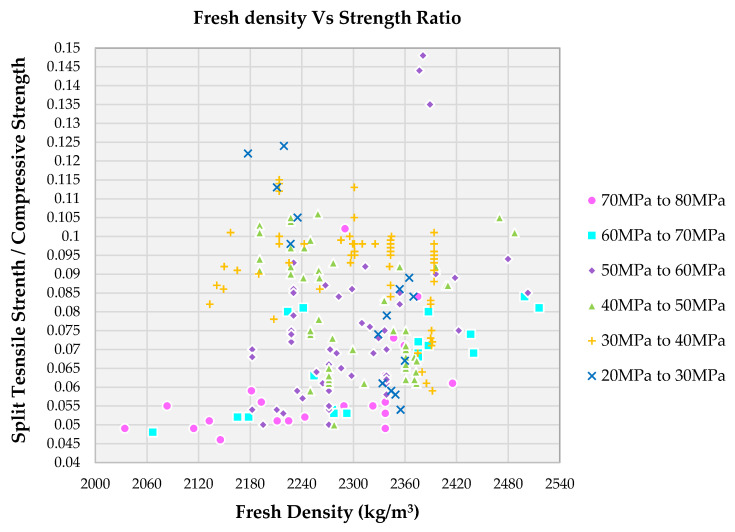
Effect of fresh density on various compressive strength of SCC concrete with recycled aggregate according to literature.

From Figure 12, it is clearly observed that family I have a lower strength ratio in the range of 0.045 to 0.060, mostly with a fresh density range of 2000 kg/m^3^ to 2400 kg/m^3^. Lower fresh density may be due to lower water content for the mix. Family II and III show a slightly higher strength ratio in the range of 0.050 to 0.095 mostly with a fresh density range of 2100 kg/m^3^ to 2500 kg/m^3^. Fresh density is decreased for family IV with an increase in strength ratio is observed. For family V and VI, the fresh density is maintained between 2100 kg/m^3^ to 2400 kg/m^3^ but the strength ratio is increased. The higher fresh density is due to an increase in the water content of the mix. The higher strength ratio has been already described earlier.

### 4.10. Impact of Compressive Load Area of RCA on Strength Ratio

The most common experiment carried out on concrete is the compressive strength experiment. Because of ease of experiment procedure and cost effectiveness, the most recommended experiment is compressive strength. As per various international standards, the most used geometrices are cyclinders with slenderness ratio as two and cubes. The effect of specimen shape on compressive strength has been studied widely and different relationships obtained for these geometrical shapes have been proposed in the literature [95]. For cube specimens, the stress resisted by the larger specimen is less compared to smaller specimens as reported in the literature [95] whereas for cylindrical specimens, the stress remains constant for smaller changes in geometry. The size effect in cubical specimens is much stronger than the cylindrical specimens. The wall effect is defined as the amount of mortar required to fill the space between concrete’s aggregates being less than the amount of mortar needed to fill the space between aggregates and the mold’s wall. The extra mortar between aggregates and the wall of molds causes an increase in compressive/split tensile strength of specimens [96].

**Figure 13:**
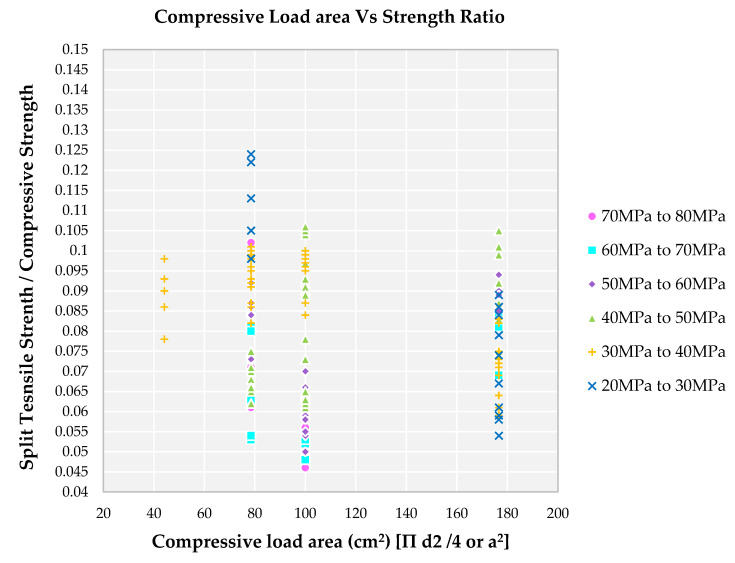
Impact of compressive load area on various compressive strength of SCC concrete with recycled aggregate according to literature.

For cylindrical specimens, when the height of a cylinder is less than the diameter of the cylinder, then the type of failure when subjected to uniaxial compression in the concrete is crushing. If the height of the cylinder is greater than the diameter of the cylinder then the type of failure when subjected to uniaxial compression is cracking. If the diameter of the cylinder is equal to the height of the cylinder, then the type of failure when subjected to uniaxial compression in concrete is mostly cracking at 45 degrees [95]. Stress resistance is mentioned in the terms of compressive load area irrespective of geometrical shape and size of specimens. Most of the families I and II use specimen size as a medium when compared to other families as observed from Figure 13. Families III and IV use larger specimen sizes and families V and VI use smaller specimen sizes as noted from Figure 13. The higher the grade of SCC, the lower the compressive load area, and the higher the compressive load area the grade of SCC [95]. Most of the compressive strength of families is done in cubes along with a size of 150 mm and next to that 100 mm of cubes are used. Size effect in uniaxial compressive strength is affected by the end restraint and energy release zone (micro crack zone, characteristics dimensions and confinement effect). The confinement effect comes into the picture when the compressive load area increases (specimen size is larger) [95]. Cylindrical specimens are used by minimum researchers to determine the compressive strength of concrete when compared to cubical specimens.

Van Vliet and van Mier., 1996 [97] determined that the shape effect is affected by the boundary friction conditions including the rotational freedom of the loading plates, and restraint at the concrete specimen-loading plate interface. For compressive strength of specimen, they concluded in the following terms (1) The compressive strength of concrete determined from specimens using conventional steel loading plates (high friction) decreases with the increase in the aspect ratio of the specimen, whereas that attained from specimens using friction dropping interlayer (low friction) in the interface is almost independent of the aspect ratio of the specimen. (2) Higher compressive strength is observed in specimens with high friction conditions than in specimens with low friction, especially with the decrease in the aspect ratio of the specimen. (3) However, the differences of compressive strength according to boundary friction condition decrease significantly as the aspect ratio of the specimen is more than 1.0 and become negligible for specimens with an aspect ratio of 2.0.

### 4.11. Impact of Split Tensile Load Area on Strength Ratio

Gonnerman (1925) experimentally exhibited that the ratio of the compressive failure stress to the compressive strength decreases as the specimen size increases. This phenomenon of reduction in strength dependent on specimen size is called the “reduction phenomenon”. When a member is subjected to pure tension loading, the failure is caused by a relatively narrowed localized zone and in the case of compression loading, the failure is caused by a larger damage zone [98]. For both cases, the failure is triggered by the distribution of splitting cracks in the direction of the length of a member as the lateral deformation increases during failure progression. The size effect of compressive failure is not as different as tension failure, because the formation of microcracks and its growth in compression failure is distributed in a longer region than in the tension failure [98].

**Figure 14:**
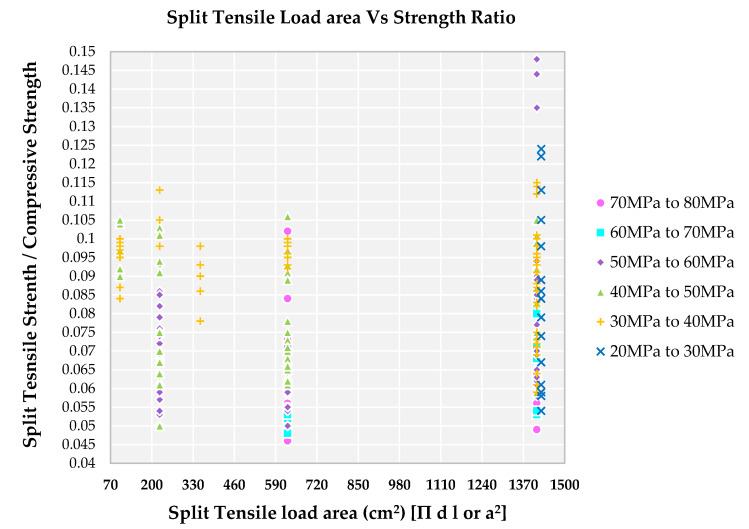
Effect of split tensile load area on various compressive strength of SCC concrete with recycled aggregate according to literature.

Cubical and cylindrical specimens are used to determine split tensile strength of concrete frequently. Various international codes across the globe contain the formula that does not consider size and shape effect in the calculation of split tensile strength of concrete. To determine the split tensile strength of concrete, cylindrical specimens are proposed by [99] and were successfully applied to cubical specimens by Nilson., 1961 [100]. Various experiments on split tensile strength specimens have revealed that the nominal strength of specimen initially decreases with an increase in specimen size and subsequently approaches constant value [101]. Smaller size specimens show higher strain when compared to that of higher size specimens, resulting in higher strength for a smaller area of specimens. Most of the results from Figure 14 indicate that the higher split tensile strength area is notified for the lower grade of SCC predominantly. Analytical and numerical studies on split-tension specimens have revealed that nominal strength is highly affected by the width of the distributed load and the specimen size [101].

The strength ratio is in the range of 0.085 to 0.105 for family IV and V, and has a lower split tensile load area due to splitting force decreasing with a decrease in the size of the specimen. A lower split tensile load area is observed for families III, IV and V from Figure 14 for strength ratio in the range of 0.05 to 0.115—this is due smaller confinement area that is required to resist the split tensile strength. Families I and II show a lower strength ratio with a higher resisting split tensile load area as observed in Figure 14. A higher split tensile load area is observed for all the families from Figure 14, with a strength ratio from 0.05 to 0.15. The higher the split tensile load area and confinement area results in the higher the ratio is, due to the total length of cracks involved in the failure process of the specimen increasing with the increase in the size of the specimen [101].

## 5. Sustainable Construction

The limited availability of natural resources and their continuous usage will lead to depletion with respect to time. To preserve natural resources, needless wasting of natural resources in any form should be restricted and regulated. Formulation and implementation of proper waste management along with appropriate knowledge on concrete properties manufactured with recycled aggregates can minimize the waste generated from C&DW. In order to promote the sustainable construction associated with using recycled aggregates in construction, some advantages are listed, such as:

- Reduction in extraction of raw materials, especially aggregates and processes associated with them [102,103]
- Reduction in the negative impact on the environment through removal and disposal processes [15]
- Introduction of circular economy [104]
- Reduction in the dependence on resources from nature [16]
- Reduction in the requirement for safe disposal sites of C&DW [103,105]
- Reduction in the cost of construction [61,106]
- Creation of new employment fields [16]
- Development of a sector based on recycled aggregates manufacturing

Accomplishment of recycling process of C&DW necessitates promotion by means of education and information in addition to legal rules from the governing body. Hence, in order to support the above, a database gathering the usage of recycle aggregates based on mechanical properties would provide concrete evidence.

## 6. Conclusions

A comprehensive review of the influence of design parameters on the strength ratio is required in the current scenario to understand the role of various ingredient proportions on strength requirements. This will help budding engineers or researchers who work on SCC with recycled aggregate as standard requirements of ingredients proportions in SCC mix.

- Increase in W/C ratio, decrease in strength of SCC (or increase in the family from I to VI). A lower W/C ratio results in a lower strength ratio for a higher family (family I, II and III).
- Increase in W/B ratio, decrease in strength of SCC (or increase in the family from I to VI). A lower W/B ratio results in a lower strength ratio for a higher family (family I, II and III). A higher W/B ratio results in a higher strength ratio for lower families (family IV, V and VI).
- FA/CA in the ratio of 1.5 to 3.0 is observed for all the families. Lower the strength ratio higher the family and higher the FA/CA ratio lower families (IV, V and VI).
- TA/C in the ratio of 3.0 to 7.0 is observed for all the families. Higher TA/C ratio, lower families (IV, V and VI) are observed for higher strength ratio. Lower the TA/C ratio, higher families (I, II and III) are observed for lower strength ratio.
- Lower strength ratio results for higher families (I, II and III) result in the usage of SP from 2.0 kg to 7.0 kg and in similar usage of SP, there is a higher strength ratio for lower families (IV, V and VI). Hence, there is no clear relationship between strength ratio and usage of SP in kg.
- W/S ratio is lower (in the range of 3.0 to 8.0) resulting in a lower strength ratio for higher families (I, II and III). Higher W/S ratio, above 9.0, results in lower families (IV, V and VI).
- Lower level of replacement of NFA by RFA is observed for all families. A 100% replacement is observed in higher families only.
- A 50% replacement of NCA by RCA is observed for all the families, showing a lesser difference in strength ratio. A 100% replacement is observed for all the families.
- Lower fresh density for higher families (I, II and III) results in a lower strength ratio and higher strength ratio is from higher fresh density for lower families (IV, V and VI).
- Higher compressive load area results in a higher strength ratio for lower families (IV, V and VI) and a lower strength ratio is from higher families (I, II and III) leading to the lower compressive load area.
- Similar to compressive load area results, the higher the split tensile strength load area, the higher the strength ratio for lower families (IV, V and VI). A lower split tensile load area, the lower the strength ratio from higher families (I, II, III).

Hence, this overall review may be helpful for researchers, scientists and academicians to develop standards for SCC with recycled aggregates based on the strength properties. It also guides budding engineers and builders to utilize different ingredients combinations impact on compressive and split tensile strength for various grades.

## Figures and Tables

**Table 1:** Family I (Compressive strength range–70 MPa to 80 MPa) design parameters according to literature.

Authors	Design Parameters											Strength (MPa)	
	W/C	W/B	TA/C	FA/CA	SP (kg)	W/S (%)	% RFA	% RCA	Fresh Density (kg/m^3^)	Compressive Load Area (cm^2^)	Split tensile Load Area (cm^2^)	Fck	Fsk
Gesoglu et al., 2015 (A) [48]	0.30	0.23	3.80	1.79	6.29	5.85	-	0	2322.8	100.0	628	77.96	4.25
	0.30	0.23	3.54	1.79	4.95	6.16	-	100	2211.6	100.0	628	68.67	3.50
Gesoglu et al., 2015 (B) [48]	0.30	0.20	4.34	2.04	7.00	5.11	-	0	2289.2	100.0	628	81.40	4.46
	0.30	0.20	4.05	2.04	6.26	5.37	-	100	2181.7	100.0	628	70.39	4.13
Gesoglu et al., 2015 (D) [48]	0.43	0.28	5.22	2.46	4.55	6.36	-	0	2243.8	100.0	628	72.47	3.75
	0.43	0.28	4.87	2.46	4.09	6.72	-	100	2132.7	100.0	628	63.89	3.24
Gesoglu et al., 2015 (E) [48]	0.30	0.23	3.80	1.79	6.29	5.85	0	-	2322.8	100.0	628	77.96	4.25
	0.30	0.23	3.57	1.79	4.51	6.12	100	-	2224.9	100.0	628	61.97	3.15
Gesoglu et al., 2015 (F) [48]	0.30	0.20	4.34	2.04	7.00	5.11	0	-	2289.2	100.0	628	81.40	4.46
	0.30	0.20	4.08	2.04	4.20	5.34	100	-	2193.2	100.0	628	64.61	3.59
Gesoglu et al., 2015 (H) [48]	0.43	0.28	5.22	2.46	4.55	6.36	0	-	2243.8	100.0	628	72.47	3.75
	0.43	0.28	4.91	2.46	2.70	6.67	100	-	2145.5	100.0	628	61.04	2.81
Gesoglu et al., 2015 (I) [48]	0.30	0.23	3.80	1.79	6.29	5.85	0	0	2322.8	100.0	628	77.96	4.25
	0.30	0.23	3.31	1.79	3.44	6.46	100	100	2114.6	100.0	628	55.76	2.72
Gesoglu et al., 2015 (J) [48]	0.30	0.20	4.34	2.04	7.00	5.11	0	0	2289.2	100.0	628	81.40	4.46
	0.30	0.20	3.79	2.04	3.79	5.64	100	100	2083.5	100.0	628	57.41	3.16
Gesoglu et al., 2015 (L) [48]	0.43	0.28	5.22	2.46	4.55	6.36	0	0	2243.8	100.0	628	72.47	3.75
	0.43	0.28	4.55	2.46	2.62	7.06	100	100	2034.4	100.0	628	52.92	2.59
Wang et al., 2020 [50]	0.36	0.35	4.37	2.309	6.50	6.64	0	-	2415.5	78.5	628	72.30	4.43
	0.36	0.35	4.23	2.174	6.50	6.81	5	-	2359.5	78.5	628	58.50	4.15
	0.36	0.35	4.27	2.174	6.50	6.77	10	-	2374.9	78.5	628	56.00	4.70
	0.36	0.35	4.20	2.174	6.50	6.85	15	-	2346.9	78.5	628	50.10	3.67
	0.36	0.35	4.07	2.174	6.50	7.03	25	-	2290.5	78.5	628	32.20	3.30
Sadeghi-Nik., et al. 2019 (B) [49]	0.41	0.34	3.84	2.180	7.00	8.44	0	0	2337.3	100.0	1413	74.10	3.60
	0.41	0.34	3.84	2.180	7.00	8.44	20	20	2337.3	100.0	1413	55.00	3.10
	0.41	0.34	3.84	2.180	7.00	8.44	40	40	2337.3	100.0	1413	52.10	2.90
	0.41	0.34	3.84	2.180	7.00	8.44	60	60	2337.3	100.0	1413	51.00	2.70
	0.41	0.34	3.84	2.180	7.00	8.44	80	80	2337.3	100.0	1413	50.50	2.70
	0.41	0.34	3.84	2.180	7.00	8.44	100	100	2337.3	100.0	1413	40.00	2.50

W/C = water to cement ratio; W/B = water to binder ratio; TA/C = total aggregate to cement ratio; FA/CA = fine aggregate to coarse aggregate ratio; SP = superplasticizer; W/S = water to solid percentage; RFA = recycled fine aggregate; RCA = recycled coarse aggregate; fck = Compressive strength; fsk = split tensile strength.

**Table 2:** Family II (Compressive strength range–60 MPa to 70 MPa) design parameters according to literature.

Authors	Design Parameters											Strength (MPa)	
	W/C	W/B	TA/C	FA/CA	SP (kg)	W/S (%)	% RFA	% RCA	Fresh Density (kg/m^3^)	Compressive Load Area (cm^2^)	Split Tensile Load Area (cm^2^)	Fck	Fsk
Gesoglu et al., 2015 (C) [48]	0.430	0.32	4.563	2.148	3.11	7.29	-	0	2322.8	78.50	1413	66.63	3.50
	0.430	0.32	4.252	2.148	2.55	7.70	-	100	2211.6	78.50	1413	55.38	2.89
Gesoglu et al., 2015 (G) [48]	0.430	0.32	4.563	2.148	3.11	7.29	0	-	2289.2	78.50	1413	66.63	3.50
	0.430	0.32	4.288	2.148	1.82	7.65	100	-	2181.7	78.50	1413	48.69	2.53
Gesoglu et al., 2015 (K) [48]	0.430	0.32	4.563	2.148	3.11	7.29	0	0	2243.8	78.50	1413	66.63	3.50
	0.430	0.32	3.978	2.148	1.78	8.10	100	100	2132.7	100.00	628	46.04	2.20
Revilla Cuesta et al., 2020 [51]	0.459	0.29	5.583	3.833	6.60	6.39	-	0	2322.8	100.00	628	60.00	3.20
	0.470	0.29	5.516	3.767	6.60	6.60	-	25	2224.9	100.00	628	56.00	3.03
	0.482	0.30	5.433	3.683	6.60	6.85	-	50	2289.2	100.00	628	44.00	2.75
	0.506	0.32	5.367	3.617	6.60	7.26	-	75	2193.2	100.00	628	31.00	2.50
	0.530	0.33	5.283	3.533	6.60	7.70	-	100	2243.8	100.00	628	30.00	2.40
Fiol et al., 2018 (C) [52]	0.310	0.17	5.625	2.031	1.80	4.13	-	0	2145.5	176.63	1413	63.36	5.30
	0.300	0.16	5.688	2.031	1.80	3.97	-	20	2322.8	176.63	1413	64.13	5.21
	0.270	0.14	5.469	2.094	2.25	3.68	-	50	2114.6	176.63	1413	66.82	4.95
	0.250	0.13	5.500	2.250	2.85	3.39	-	100	2289.2	176.63	1413	72.81	5.00
Behera et al., 2019 (A) [53]	0.560	0.34	5.350	2.960	5.23	7.98	0	-	2083.5	225.00	1413	60.76	4.13
	0.560	0.34	5.350	2.960	5.23	7.98	50	-	2243.8	225.00	1413	55.76	3.82
	0.560	0.34	5.350	2.960	5.23	7.98	100	-	2034.4	225.00	1413	44.54	3.20
Behera et al., 2019 (B) [53]	0.560	0.34	5.350	2.960	5.23	7.98	0	-	2415.5	225.00	1413	60.76	4.13
	0.560	0.34	5.350	2.960	3.15	7.98	0	-	2359.5	225.00	1413	44.54	3.56
	0.560	0.34	5.350	2.960	4.20	7.98	100	-	2374.9	225.00	1413	38.41	2.71

W/C = water to cement ratio; W/B = water to binder ratio; TA/C = total aggregate to cement ratio; FA/CA = fine aggregate to coarse aggregate ratio; SP = superplasticizer; W/S = water to solid percentage; RFA = recycled fine aggregate; RCA = recycled coarse aggregate; fck = Compressive strength; fsk = split tensile strength.

**Table 3:** Family III (Compressive strength range–50 MPa to 60 MPa) design parameters according to literature.

Authors	Design Parameters											Strength (MPa)	
	W/C	W/B	TA/C	FA/CA	SP (kg)	W/S (%)	% RFA	% RCA	Fresh Density (kg/m^3^)	Compressive Load Area (cm^2^)	Split Tensile Load Area (cm^2^)	Fck	Fsk
Señas et al., 2016 (B) [56]	0.40	0.40	4.373	2.566	4.15	7.44	0	0	2395.8	176.63	1413	51.20	4.60
	0.40	0.40	4.272	2.566	4.98	7.59	0	50	2353.9	176.63	1413	48.10	4.10
	0.40	0.40	4.229	2.523	5.81	7.65	50	50	2336.0	176.63	1413	45.60	3.80
Aslani et al., 2018 (C) [54]	1.13	0.45	9.311	5.090	3.75	9.57	0	0	2329.4	78.50	1413	50.39	3.70
	1.13	0.45	9.225	5.050	2.95	9.64	10	20	2313.9	78.50	1413	47.74	4.38
	1.13	0.45	9.139	5.010	3.55	9.71	20	20	2298.4	78.50	1413	46.06	3.98
	1.13	0.45	9.054	4.960	3.75	9.78	30	20	2283.1	78.50	1413	45.13	3.79
	1.13	0.45	8.968	4.920	3.95	9.85	40	20	2267.6	78.50	1413	43.82	3.80
Guo et al., 2020 [55]	0.35	0.35	3.177	1.510	4.02	8.38	-	0	2354.0	225.00	225	53.45	4.39
	0.35	0.35	3.143	1.510	4.16	8.45	-	50	2336.4	225.00	225	46.54	3.49
	0.35	0.35	3.110	1.510	4.72	8.52	-	100	2319.2	225.00	225	43.89	3.32
	0.35	0.18	6.219	3.019	4.78	4.26	-	100	2227.9	225.00	225	21.00	1.57
	0.35	0.18	6.219	3.019	5.67	4.26	-	100	2227.9	225.00	225	38.38	2.75
	0.35	0.18	6.219	3.019	6.86	4.26	-	100	2227.9	225.00	225	49.44	3.66
	0.35	0.09	12.438	6.038	7.40	2.13	-	100	2182.4	225.00	225	13.64	0.96
	0.35	0.09	12.438	6.038	7.28	2.13	-	100	2182.4	225.00	225	30.84	1.67
	0.35	0.09	12.438	6.038	7.57	2.13	-	100	2182.4	225.00	225	42.75	2.89
Fiol et al., 2018 (B) [52]	0.31	0.15	6.207	2.241	1.80	3.76	-	0	2479.9	176.63	1413	58.30	5.50
	0.32	0.16	6.276	2.241	1.80	3.85	-	20	2502.8	176.63	1413	60.25	5.15
	0.27	0.13	6.034	2.310	2.25	3.35	-	50	2418.2	176.63	1413	58.52	5.20
	0.25	0.12	6.069	2.483	2.85	3.09	-	100	2422.5	176.63	1413	70.56	5.32
Uygunoglu et al., 2014 [57]	0.31	0.24	4.874	2.057	10.80	5.03	-	0	2264.4	225.00	225	57.00	3.50
	0.31	0.24	4.920	2.057	10.80	5.00	-	100	2280.5	225.00	225	54.00	3.70
	0.34	0.26	4.777	2.017	9.50	5.61	-	0	2241.0	225.00	225	56.60	3.20
	0.34	0.26	4.823	2.017	9.50	5.57	-	100	2257.1	225.00	225	53.20	3.40
	0.37	0.29	4.683	1.977	8.10	6.20	-	0	2218.6	225.00	225	56.10	3.00
	0.37	0.29	4.729	1.977	8.10	6.15	-	100	2234.7	225.00	225	52.50	3.10
	0.40	0.31	4.586	1.937	6.80	6.81	-	0	2195.1	225.00	225	55.60	2.80
	0.40	0.31	4.631	1.937	6.80	6.76	-	100	2210.9	225.00	225	51.70	2.80
Kou et al., 2009 (B) [58]	0.44	0.25	4.447	1.947	8.50	7.05	0	-	2271.6	100.00	628	53.70	2.90
	0.44	0.25	4.447	1.888	8.50	7.05	25	-	2271.6	100.00	628	64.30	3.20
	0.44	0.25	4.447	1.829	8.50	7.05	50	-	2271.6	100.00	628	62.30	3.40
	0.44	0.25	4.447	1.771	8.50	7.05	75	-	2271.6	100.00	628	56.30	3.30
	0.44	0.25	4.447	1.709	8.50	7.05	100	-	2271.6	100.00	628	53.20	3.50
Sadeghi-Nik., et al. 2019 (B) [49]	0.41	0.34	3.843	2.176	7.00	8.10	0	0	2338.6	100.00	1413	52.00	3.30
	0.41	0.34	3.843	2.176	7.00	8.10	20	20	2338.6	100.00	1413	43.10	2.70
	0.41	0.34	3.843	2.176	7.00	8.10	40	40	2338.6	100.00	1413	41.00	2.50
	0.41	0.34	3.843	2.176	7.00	8.10	60	60	2338.6	100.00	1413	40.10	2.50
	0.41	0.34	3.843	2.176	7.00	8.10	80	80	2338.6	100.00	1413	38.10	2.20
	0.41	0.34	3.843	2.176	7.00	8.10	100	100	2338.6	100.00	1413	30.00	2.10
Grdic et al., 2010 [59]	0.42	0.25	3.748	2.098	4.00	7.80	-	0	2376.8	225.00	1413	50.00	7.20
	0.43	0.27	3.748	2.098	4.00	7.99	-	50	2380.9	225.00	1413	48.00	7.10
	0.45	0.28	3.748	2.098	4.00	8.36	-	100	2389.1	225.00	1413	46.00	6.20
Tang et al., 2016 [60]	0.49	0.35	3.315	1.831	4.50	10.36	-	0	2323.3	225.00	1413	59.00	4.10
	0.49	0.35	3.285	1.831	4.50	10.43	-	25	2309.9	225.00	1413	64.00	4.90
	0.49	0.35	3.258	1.831	4.50	10.49	-	50	2297.9	225.00	1413	65.00	4.10
	0.49	0.35	3.231	1.831	4.50	10.55	-	75	2285.9	225.00	1413	60.00	3.90
	0.49	0.35	3.202	1.831	4.50	10.61	-	100	2273.0	225.00	1413	54.00	3.80
Tuyan et al., 2014 (A) [61]	0.43	0.32	4.708	2.365	4.90	6.47	-	0	2230.5	225.00	225	52.30	4.86
	0.43	0.32	4.708	2.365	5.50	6.47	-	20	2230.5	225.00	225	54.70	4.69
	0.43	0.32	4.708	2.365	5.70	6.47	-	40	2230.5	225.00	225	57.20	4.51
	0.43	0.32	4.708	2.365	6.20	6.47	-	60	2230.5	225.00	225	51.10	4.33

W/C = water to cement ratio; W/B = water to binder ratio; TA/C = total aggregate to cement ratio; FA/CA = fine aggregate to coarse aggregate ratio; SP = superplasticizer; W/S = water to solid percentage; RFA = recycled fine aggregate; RCA = recycled coarse aggregate; fck = Compressive strength; fsk = split tensile strength.

**Table 4:** Family IV (Compressive strength range–40 MPa to 50 MPa) design parameters according to literature.

Authors	Design Parameters											Strength (MPa)	
	W/C	W/B	TA/C	FA/CA	SP (kg)	W/S (%)	% RFA	% RCA	Fresh Density (kg/m^3^)	Compressive Load Area (cm^2^)	Split Tensile Load Area (cm^2^)	Fck	Fsk
Señas et al., 2016 (A) [56]	0.40	0.40	4.37	2.57	2.91	7.44	0	0	2395.8	176.63	1413	47.60	4.40
	0.40	0.40	4.27	2.57	5.40	7.59	0	50	2353.9	176.63	1413	46.60	4.30
	0.40	0.40	4.23	2.52	6.23	7.65	50	50	2336.0	176.63	1413	47.00	3.90
Martínez-García et al., 2020 [19]	0.47	0.41	4.01	2.26	3.20	9.12	-	0	2250.0	176.63	1413	46.36	4.60
	0.47	0.41	4.01	2.26	4.00	9.12	-	25	2250.0	176.63	1413	55.58	4.10
	0.47	0.41	4.01	2.26	4.80	9.12	-	50	2250.0	176.63	1413	54.70	3.20
	0.47	0.41	4.01	2.26	5.40	9.12	-	100	2250.0	176.63	1413	44.04	3.30
Fiol et al., 2018 (A) [52]	0.40	0.18	7.20	2.60	1.80	4.22	-	0	2470.0	176.63	1413	49.09	5.17
	0.39	0.17	7.28	2.60	1.80	4.08	-	20	2487.5	176.63	1413	49.98	5.06
	0.35	0.15	7.00	2.68	2.25	3.77	-	50	2407.5	176.63	1413	55.64	4.85
	0.32	0.14	7.04	2.88	2.85	3.43	-	100	2410.0	176.63	1413	56.75	4.92
Duan et al., 2020 (A) [64]	0.40	0.28	3.35	1.53	1.53	8.38		0	2227.2	100.00	100	42.91	4.45
	0.40	0.28	3.35	1.53	1.53	8.38		0	2227.2	100.00	100	42.41	4.44
	0.40	0.28	3.35	1.53	1.53	8.38		25	2227.2	100.00	100	40.45	3.64
	0.40	0.28	3.35	1.53	1.53	8.38		50	2227.2	100.00	100	36.93	3.60
	0.40	0.28	3.35	1.53	1.53	8.38		100	2227.2	100.00	100	34.85	3.20
Duan et al., 2020 (B) [64]	0.40	0.28	3.35	1.53	1.53	8.38		0	2227.2	100.00	100	42.91	4.45
	0.40	0.28	3.35	1.53	1.53	8.38		0	2227.2	100.00	100	42.41	4.44
	0.40	0.28	3.35	1.53	1.53	8.38		25	2227.2	100.00	100	40.45	3.64
	0.40	0.28	3.35	1.53	1.53	8.38		50	2227.2	100.00	100	36.93	3.60
	0.40	0.28	3.35	1.53	1.53	8.38		100	2227.2	100.00	100	34.85	3.20
Pan et al., 2019 [65]	0.41	0.33	3.90	1.81	1.16	7.96	-	30	2370.8	225.00	225	45.60	3.10
	0.46	0.33	4.33	2.01	1.16	8.04	-	30	2373.7	225.00	225	49.00	3.30
	0.52	0.33	4.88	2.26	1.16	8.07	-	30	2373.4	225.00	225	42.53	2.60
	0.59	0.33	5.58	2.59	1.16	8.01	-	30	2371.9	225.00	225	37.22	2.30
	0.70	0.33	6.57	3.05	1.16	8.07	-	30	2371.1	225.00	225	32.11	2.00
	0.83	0.33	7.81	3.62	1.16	8.05	-	30	2372.9	225.00	225	28.10	1.80
Kou et al., 2009 (A) [66]	0.44	0.25	4.45	1.95	8.50	7.05	0	-	2271.6	100.00	628	44.30	2.90
	0.44	0.25	4.45	1.89	8.50	7.05	25	-	2271.6	100.00	628	44.50	2.70
	0.44	0.25	4.45	1.83	8.50	7.05	50	-	2271.6	100.00	628	43.40	2.70
	0.44	0.25	4.45	1.77	8.50	7.05	75	-	2271.6	100.00	628	41.30	2.60
	0.44	0.25	4.45	1.71	8.50	7.05	100	-	2271.6	100.00	628	38.70	2.50
Manzi et al., 2017 [62]	0.51	0.31	4.57	1.66	4.90	8.23	0	0	2346.6	225.00	225	43.80	3.30
	0.49	0.30	4.45	2.56	5.25	8.06	12	13	2299.4	225.00	225	45.40	3.20
	0.49	0.30	4.39	2.43	5.25	8.14	19	21	2277.7	225.00	225	50.30	2.50
	0.51	0.31	4.47	2.79	5.60	8.36	0	40	2312.7	225.00	225	51.10	3.10
Nili et al., 2019 (A) [63]	0.44	0.31	3.61	2.80	3.34	8.79	0	0	2275.8	100.00	628	46.30	3.40
	0.44	0.31	3.57	2.78	3.89	8.86	0	100	2258.9	100.00	628	32.10	3.40
	0.44	0.31	3.57	2.78	3.89	8.86	50	0	2259.7	100.00	628	35.20	3.20
	0.44	0.32	3.53	2.75	3.89	8.94	50	100	2241.9	100.00	628	38.20	3.40
Nili et al., 2019 (B) [63]	0.44	0.31	3.61	2.81	3.34	8.79	0	0	2275.8	100.00	628	46.30	3.40
	0.48	0.32	3.91	3.04	3.47	8.83	0	0	2276.7	100.00	628	46.40	4.30
	0.48	0.32	3.87	3.01	3.85	8.91	0	100	2259.9	100.00	628	52.90	4.10
	0.48	0.32	3.88	3.01	4.24	8.90	50	0	2260.7	100.00	628	32.50	2.90
	0.48	0.32	3.83	2.98	4.62	8.98	50	100	2242.9	100.00	628	31.80	3.10
Tuyan et al., 2014 (B) [61]	0.48	0.36	4.55	2.26	3.30	7.41	-	0	2191.1	225.00	225	42.20	4.33
	0.48	0.36	4.55	2.26	3.40	7.41	-	20	2191.1	225.00	225	44.00	4.44
	0.48	0.36	4.55	2.26	3.50	7.41	-	40	2191.1	225.00	225	44.30	4.01
	0.48	0.36	4.55	2.26	3.70	7.41	-	60	2191.1	225.00	225	40.90	3.85
Singh et al., 2019 (A) [1]	0.57	0.40	3.49	1.88	1.23	11.63	-	0	2361.0	78.50	628	42.34	2.62
	0.57	0.40	3.49	1.88	1.23	11.63	-	50	2361.0	78.50	628	36.49	2.49
	0.57	0.40	3.49	1.88	1.23	11.63	-	100	2361.0	78.50	628	31.53	2.37
Singh et al., 2019 (B) [1]	0.57	0.40	3.49	1.88	1.23	11.63	-	0	2361.0	78.50	628	42.34	2.62
	0.57	0.40	3.49	1.88	1.23	11.63	-	50	2361.0	78.50	628	39.42	2.55
	0.57	0.40	3.49	1.88	1.23	11.63	-	100	2361.0	78.50	628	35.08	2.46
Singh et al., 2019 (C) [1]	0.57	0.40	3.49	1.88	1.23	11.63	-	0	2361.0	78.50	628	42.34	2.62
	0.57	0.40	3.49	1.88	1.23	11.63	-	50	2361.0	78.50	628	38.69	2.54
	0.57	0.40	3.49	1.88	1.23	11.63	-	100	2361.0	78.50	628	33.88	2.42
Singh et al., 2019 (D) [1]	0.57	0.40	3.49	1.88	1.23	11.63	-	0	2361.0	78.50	628	42.34	2.62
	0.57	0.40	3.49	1.88	1.23	11.63	-	50	2361.0	78.50	628	41.85	2.61
	0.57	0.40	3.49	1.88	1.23	11.63	-	100	2361.0	78.50	628	38.02	2.52

W/C = water to cement ratio; W/B = water to binder ratio; TA/C = total aggregate to cement ratio; FA/CA = fine aggregate to coarse aggregate ratio; SP = superplasticizer; W/S = water to solid percentage; RFA = recycled fine aggregate; RCA = recycled coarse aggregate; fck = Compressive strength; fsk = split tensile strength.

**Table 5:** Family V (Compressive strength range–30 MPa to 40 MPa) design parameters according to literature.

Authors	Design Parameters											Strength (MPa)	
	W/C	W/B	TA/C	FA/CA	SP (kg)	W/S (%)	% RFA	% RCA	Fresh Density (kg/m^3^)	Compressive Load Area (cm^2^)	Split Tensile Load Area (cm^2^)	Fck	Fsk
Aslani et al., 2018 (B) [54]	1.13	0.45	8.40	5.09	4.40	10.37	0	0	2165.2	78.50	1413.00	38.93	3.54
	1.13	0.45	8.36	5.05	3.95	10.41	10	20	2157.3	78.50	1413.00	38.36	3.89
	1.13	0.45	8.31	5.01	4.15	10.45	20	20	2149.2	78.50	1413.00	39.85	3.43
	1.13	0.45	8.27	4.96	4.35	10.50	30	20	2141.3	78.50	1413.00	37.68	3.27
	1.13	0.45	8.22	4.92	4.55	10.54	40	20	2133.2	78.50	1413.00	40.68	3.34
Bahrami et al., 2020 (A) [12]	0.41	0.33	4.33	3.00	3.80	7.35	-	-	2394.0	225.00	1413.00	38.99	3.61
	0.41	0.33	4.33	3.00	3.80	7.35	25	-	2394.0	225.00	1413.00	36.70	3.45
	0.41	0.33	4.33	3.00	3.80	7.35	50	-	2394.0	225.00	1413.00	32.80	3.22
	0.41	0.33	4.33	3.00	3.80	7.35	75	-	2394.0	225.00	1413.00	29.60	2.70
	0.41	0.33	4.33	3.00	3.80	7.35	100	-	2394.0	225.00	1413.00	24.80	2.50
Bahrami et al., 2020 (B) [12]	0.41	0.33	4.33	3.00	3.80	7.35	-	-	2394.0	225.00	1413.00	38.99	3.61
	0.41	0.33	4.33	3.00	3.80	7.35	-	25	2394.0	225.00	1413.00	37.00	3.55
	0.41	0.33	4.33	3.00	3.80	7.35	-	50	2394.0	225.00	1413.00	33.51	3.38
	0.41	0.33	4.33	3.00	3.80	7.35	-	75	2394.0	225.00	1413.00	31.36	2.76
	0.41	0.33	4.33	3.00	3.80	7.35	-	100	2394.0	225.00	1413.00	25.83	2.46
Silva et al., 2016 [34]	0.45	0.36	4.19	2.51	2.88	8.28	-	0	2261.0	44.16	353.25	35.50	3.05
	0.45	0.36	4.14	2.51	2.88	8.35	-	25	2242.9	44.16	353.25	27.69	2.70
	0.45	0.36	4.10	2.51	2.89	8.42	-	50	2225.3	44.16	353.25	29.11	2.71
	0.45	0.36	4.10	2.51	2.89	8.49	-	75	2207.6	44.16	353.25	30.18	2.35
	0.45	0.36	4.00	2.51	2.90	8.57	-	100	2190.0	44.16	353.25	25.21	2.28
Sun et al., 2020 (A) [68]	0.40	0.32	4.03	1.95	2.40	7.58	0	0	2344.4	100.00	100.00	40.26	4.03
	0.40	0.32	4.03	1.95	2.42	7.58	10	0	2343.4	100.00	100.00	39.05	3.28
	0.40	0.32	4.03	1.95	2.44	7.58	10	25	2343.4	100.00	100.00	38.04	3.32
	0.40	0.32	4.03	1.95	2.46	7.58	10	50	2343.4	100.00	100.00	35.46	3.37
	0.40	0.32	4.03	1.95	2.48	7.58	10	100	2343.4	100.00	100.00	33.35	3.28
Sun et al., 2020 (B) [68]	0.40	0.32	4.03	1.95	2.40	7.58	0	0	2344.4	100.00	100.00	40.26	4.03
	0.40	0.32	4.03	1.95	2.62	7.58	10	0	2343.4	100.00	100.00	35.36	3.41
	0.40	0.32	4.03	1.95	2.85	7.58	10	25	2343.4	100.00	100.00	35.58	3.53
	0.40	0.32	4.03	1.95	3.28	7.58	10	50	2343.4	100.00	100.00	32.63	3.17
	0.40	0.32	4.03	1.95	3.72	7.58	10	100	2343.4	100.00	100.00	32.39	2.81
Surendar et al., 2021 [69]	0.45	0.45	3.43	1.81	1.90	10.16	-	0	1854.0	225.00	1413.00	36.66	4.12
	0.45	0.45	4.38	1.81	1.90	8.37	-	10	2213.9	225.00	1413.00	36.22	4.07
	0.45	0.45	4.38	1.81	1.90	8.37	-	15	2213.9	225.00	1413.00	35.77	4.00
	0.45	0.45	4.38	1.81	1.90	8.37	-	20	2213.9	225.00	1413.00	34.95	3.98
	0.45	0.45	4.38	1.81	1.90	8.37	-	25	2213.9	225.00	1413.00	34.50	3.96
	0.45	0.45	4.38	1.81	1.90	8.37	-	50	2213.9	225.00	1413.00	32.44	3.23
	0.45	0.45	4.38	1.81	1.90	8.37	-	75	2213.9	225.00	1413.00	27.60	2.70
Tuyan et al., 2014 (A) [61]	0.53	0.4	4.87	2.18	2.20	7.83	-	0	2301.1	225.00	225.00	37.20	4.19
	0.53	0.4	4.87	2.18	2.40	7.83	-	20	2301.1	225.00	225.00	37.70	3.95
	0.53	0.4	4.87	2.18	2.70	7.83	-	40	2301.1	225.00	225.00	38.20	3.73
	0.53	0.4	4.87	2.18	3.00	7.83	-	60	2301.1	225.00	225.00	35.80	3.52
Babalolaa et al., 2020 (A) [67]	0.45	0.45	3.52	1.23	3.46	9.97	-	0	2149.9	78.50	628.00.	30.60	2.80
	0.81	0.45	11.78	4.81	2.30	5.97	-	100	2301.6	78.50	628.00	31.70	3.00
	0.90	0.45	13.09	5.35	2.30	5.97	-	100	2301.6	78.50	628.00	33.50	3.20
	1.01	0.45	14.84	6.06	2.29	5.91	-	100	2299.4	78.50	628.00	35.80	3.50
	1.15	0.45	16.98	6.94	2.28	5.88	-	100	2297.7	78.50	628.00	37.00	3.52
	1.36	0.45	20.05	8.19	2.27	5.89	-	100	2296.8	78.50	628.00	39.80	3.70
	1.64	0.45	24.17	9.87	2.26	5.90	-	100	2295.9	78.50	628.00	39.20	3.91
Babalolaa et al., 2020 (B) [67]	0.45	0.45	3.52	1.23	3.46	9.97	-	0	2149.9	78.50	628.00	30.60	2.80
	0.81	0.45	11.78	4.81	2.30	5.97	-	100	2301.6	78.50	628.00	31.70	3.00
	0.66	0.37	11.93	4.87	2.82	4.66	-	100	2342.2	78.50	628.00	41.40	3.82
	0.81	0.35	13.46	5.50	2.62	5.13	-	100	2325.4	78.50	628.00	38.00	3.74
	0.98	0.40	15.20	6.21	2.43	5.55	-	100	2310.5	78.50	628.00	37.40	3.68
	1.15	0.45	16.98	6.94	2.28	5.88	-	100	2297.7	78.50	628.00	37.00	3.52
	1.34	0.50	19.04	7.78	2.14	6.16	-	100	2285.7	78.50	628.00	33.00	3.28
Nieto et al., 2019 (C) [70]	0.45	0.35	4.11	2.33	6.10	8.34	-	0	2390.0	176.63	1413.00	38.78	2.82
	0.45	0.35	4.09	2.33	6.10	8.36	-	20	2385.0	176.63	1413.00	43.01	2.63
	0.45	0.35	4.08	2.33	6.10	8.38	-	40	2380.0	176.63	1413.00	44.96	2.88
	0.45	0.35	4.07	2.33	6.10	8.40	-	60	2375.0	176.63	1413.00	47.69	3.27
Nieto et al., 2019 (D) [70]	0.45	0.35	4.11	2.33	6.10	8.34	-	0	2390.0	176.63	1413.00	38.78	2.82
	0.46	0.36	4.09	2.33	6.10	8.59	-	20	2390.0	176.63	1413.00	40.09	3.33
	0.48	0.37	4.08	2.33	6.10	8.88	-	40	2391.0	176.63	1413.00	41.30	2.95
	0.49	0.38	4.07	2.33	6.10	9.17	-	60	2392.0	176.63	1413.00	40.54	2.40
Nieto et al., 2019 (E) [70]	0.45	0.35	4.11	2.33	6.10	8.34	-	0	2390.0	176.63	1413.00	38.78	2.82
	0.46	0.36	4.09	2.33	6.10	8.59	-	20	2390.0	176.63	1413.00	40.78	3.34
	0.48	0.37	4.08	2.33	6.10	8.88	-	40	2391.0	176.63	1413.00	43.07	3.24
	0.49	0.38	4.07	2.33	6.10	9.17	-	60	2392.0	176.63	1413.00	42.94	3.08

W/C = water to cement ratio; W/B = water to binder ratio; TA/C = total aggregate to cement ratio; FA/CA = fine aggregate to coarse aggregate ratio; SP = superplasticizer; W/S = water to solid percentage; RFA = recycled fine aggregate; RCA = recycled coarse aggregate; fck = Compressive strength; fsk = split tensile strength.

**Table 6:** Family VI (Compressive strength range–20 MPa to 30 MPa) design parameters according to literature.

Authors	Design Parameters											Strength (MPa)	
	W/C	W/B	TA/C	FA/CA	SP (kg)	W/S (%)	% RFA	% RCA	Fresh Density (kg/m^3^)	Compressive Load Area (cm^2^)	Split Tensile Load Area (cm^2^)	Fck	Fsk
Aslani et al., 2018 (A) [54]	1.13	0.45	8.47	5.09	3.35	10.30	0	0	2177.6	78.50	1426.5	22.21	2.71
	1.13	0.45	8.79	5.05	4.35	10.01	10	20	2235.2	78.50	1426.5	28.63	3.02
	1.13	0.45	8.74	5.01	4.75	10.05	20	20	2227.1	78.50	1426.5	28.01	2.75
	1.13	0.45	8.69	4.96	4.95	10.09	30	20	2219.2	78.50	1426.5	24.03	2.97
	1.13	0.45	8.65	4.92	5.35	10.13	40	20	2211.1	78.50	1426.5	27.13	3.07
Nieto et al., 2019 (A) [70]	0.55	0.42	4.56	2.59	5.50	9.39	-	0	2354.0	176.63	1426.5	25.11	2.15
	0.55	0.42	4.55	2.59	5.50	9.41	-	20	2349.0	176.63	1426.5	27.65	1.61
	0.55	0.42	4.54	2.59	5.50	9.43	-	40	2344.0	176.63	1426.5	35.86	2.12
	0.55	0.42	4.52	2.59	5.50	9.45	-	60	2339.0	176.63	1426.5	29.20	2.31
	0.55	0.42	4.51	2.59	5.50	9.47	-	80	2334.0	176.63	1426.5	34.29	2.10
	0.55	0.42	4.50	2.59	5.50	9.50	-	100	2329.0	176.63	1426.5	34.17	2.52
Nieto et al., 2019 (B) [70]	0.50	0.38	4.34	2.46	5.80	8.87	-	0	2370.0	176.63	1426.5	24.78	2.07
	0.50	0.38	4.33	2.46	5.80	8.89	-	20	2365.0	176.63	1426.5	31.25	2.78
	0.50	0.38	4.31	2.46	5.80	8.91	-	40	2360.0	176.63	1426.5	40.69	2.74
	0.50	0.38	4.30	2.46	5.80	8.93	-	60	2355.0	176.63	1426.5	38.56	2.08

W/C = water to cement ratio; W/B = water to binder ratio; TA/C = total aggregate to cement ratio; FA/CA = fine aggregate to coarse aggregate ratio; SP = superplasticizer; W/S = water to solid percentage; RFA = recycled fine aggregate; RCA = recycled coarse aggregate; fck = Compressive strength; fsk = split tensile strength.

## Data Availability

Not applicable.
